# Farm and Companion Animal Organoid Models in Translational Research: A Powerful Tool to Bridge the Gap Between Mice and Humans

**DOI:** 10.3389/fmedt.2022.895379

**Published:** 2022-05-12

**Authors:** Minae Kawasaki, Takashi Goyama, Yurika Tachibana, Itsuma Nagao, Yoko M. Ambrosini

**Affiliations:** Department of Veterinary Clinical Sciences, College of Veterinary Medicine, Washington State University, Pullman, WA, United States

**Keywords:** animal organoid, comparative medicine, One Health, public health, translational research

## Abstract

Animal organoid models derived from farm and companion animals have great potential to contribute to human health as a One Health initiative, which recognize a close inter-relationship among humans, animals and their shared environment and adopt multi-and trans-disciplinary approaches to optimize health outcomes. With recent advances in organoid technology, studies on farm and companion animal organoids have gained more attention in various fields including veterinary medicine, translational medicine and biomedical research. Not only is this because three-dimensional organoids possess unique characteristics from traditional two-dimensional cell cultures including their self-organizing and self-renewing properties and high structural and functional similarities to the originating tissue, but also because relative to conventional genetically modified or artificially induced murine models, companion animal organoids can provide an excellent model for spontaneously occurring diseases which resemble human diseases. These features of companion animal organoids offer a paradigm-shifting approach in biomedical research and improve translatability of *in vitro* studies to subsequent *in vivo* studies with spontaneously diseased animals while reducing the use of conventional animal models prior to human clinical trials. Farm animal organoids also could play an important role in investigations of the pathophysiology of zoonotic and reproductive diseases by contributing to public health and improving agricultural production. Here, we discuss a brief history of organoids and the most recent updates on farm and companion animal organoids, followed by discussion on their potential in public health, food security, and comparative medicine as One Health initiatives. We highlight recent evolution in the culturing of organoids and their integration with organ-on-a-chip systems to overcome current limitations in *in vitro* studies. We envision multidisciplinary work integrating organoid culture and organ-on-a-chip technology can contribute to improving both human and animal health.

## Introduction

Organoids are three-dimensional (3D) cellular structures that possess self-organizing and self-renewing properties, resembling the structures and functions of the originating donor organs (1, 2). Organoids can be generated from adult stem cells (ASCs) (3, 4) or pluripotent stem cells (PSCs) such as embryonic stem cells (ESCs) (5, 6) and induced pluripotent stem cells (iPSCs) (7, 8). Both ASCs and ESCs are undifferentiated cells that are either present among differentiated cells in a tissue or organ or derived from the inner cell mass of blastocysts, respectively (9). iPSCs are derived from differentiated somatic cells which are converted to regain pluripotency (9). While ASCs can only differentiate into distinct cell types of their original tissue, ESCs and iPSCs can differentiate into all cell types within the body (9). Further, organoids can be generated from tissue samples from both healthy and diseased animals. Thus, they can effectively model both normal and pathological conditions in *in vitro* environment (10–12). At present, development of farm and companion animal organoids has only been reported using ASCs (2). In this paper, the term farm animals refers to common production animals in agriculture such as pig, cattle, sheep, horses and chickens. The term companion animals refers to common domesticated animal species that have close daily relationship with humans (13) such as dogs, cats, and rabbits.

Although many studies have been conducted on organoids in humans and mice (5, 14, 15), studies focusing on organoids in farm and companion animal species have been gaining attention in various fields including veterinary and translational medicine as well as biomedical research. This is because companion animals that develop spontaneous chronic disorders similar to those of humans have been suggested to serve as better models of human patients than traditional murine models where the disorders are usually genetically or chemically induced (16, 17). This is a paradigm-shifting approach in biomedical research following on sometimes poor predictive values of the preclinical murine disease models for human clinical trials (16). Natural disease models of companion animal organoids possess great potential in *in vitro* to *in vivo* translatability, which can complement human organoid studies through their application in comparative medicine. Studying zoonotic diseases in farm animals have also gained promise in contributing to human health because many of them serve as key reservoirs for infectious diseases which result in significant morbidity and mortality in humans (1). Farm animal organoids can offer a useful model to investigate mechanisms of disease development and host defense mechanisms, which are relevant for achieving improved management strategies against pathogens with public health concerns. Additionally, relevance of farm animal organoids to human health is extended to the field of agricultural productivity through improved animal health, feed efficiency and reproductive success, which can contribute to food security to support an ever growing world population.

This multi- and trans-disciplinary approach to achieve optimum health outcomes in both humans and animals has become more popularly referred to as One Health, which recognizes that the health of humans, animals and the environment are closely linked to each other (18–20). While One Health is not a new concept for zoonotic diseases and food security, application of this initiative to naturally occurring chronic conditions between animals and humans has gained more attentions in order to achieve better predictive values during preclinical studies (16). One Health initiatives focusing on research that could benefit both veterinary and human medicine could greatly improve relevance and efficacy of *in vitro* studies in translational and biomedical research in humans (18, 19). Studying farm and companion animal organoid models enables detailed mechanistic investigation of normal physiology and development of organs and tissues (11, 21, 22), mechanisms of diseases (23–25), toxicity and drug efficacy testing (26–28), and investigation of novel diagnostics and therapeutics (3, 29–31), which all could play an important role in bridging a gap between conventional murine models and humans. Animal organoids derived from different species would provide an effective tool to evaluate cross-species variations in disease susceptibility or species-specific infectivity of zoonotic and zooanthroponotic pathogens, providing a great alternative to animal models for infection studies.

In this paper, we review a brief history of organoids and recent advances in organoid culture systems in different animal species, focusing mainly on farm animals which carry significant economic and public health impacts and companion animals which naturally develop spontaneous diseases similar to humans. We also describe key roles that farm and companion animal organoids can play in the context of One Health initiatives with a particular focus in their potential applications to comparative medicine as well as in their relevance to public health and food security in agricultural production animals. We also describe advantages and limitations of 3D organoid culture systems and different culture platforms to overcome some of these limitations such as organoid-derived two-dimensional (2D) monolayer culture systems and organ-on-a-chip technology. Finally, we discuss future perspectives of multidisciplinary work integrating organoid culture and organ-on-a-chip technology in veterinary and translational medicine.

## Development Of Farm and Companion Animal Organoid Models: History and Recent Updates

### A Brief History From Cell Aggregates to 3D Models

While studies of organoids have grown dramatically during the past decade, the history of 3D organoid cultures started as early as 1907 when self-organizing and regenerating capacity of dissociated sponge cells were demonstrated for the first time (32). Later, successful generation of different types of organs from dissociated amphibian pronephros (33) and chick embryos (34) were described in 1944 and 1960, respectively. These results highlighted the importance of internal self-organization rather than external induction in the process of organogenesis. In 1981, isolation and establishment of PSCs from mouse embryos were reported for the first time (35, 36). Following this study, isolation and culturing of human PSCs were first achieved from blastocysts in 1998 (37). These studies pioneered stem cell research, together with subsequent studies which reported establishment of iPSCs from reprogrammed mouse and human fibroblasts (38, 39) and human somatic cells (40) in mid-2000s.

As described earlier, organoids can be generated from ASCs (3, 4), ESCs (5, 6), or iPSCs (7, 8). Development of 3D organoids began to increase in the late 2000s and early 2020s. Two landmark studies reported generation of cerebral cortex tissue from mouse and human ESCs (41) and intestinal organoids from a single intestinal mouse ASC (42). Although murine and human organoids remain to be the most extensively investigated models, organoids of other animal species have been gaining more attention in recent years including farm and companion animals. Supplementary Table 1 provides a summary of previous studies related to 3D organoids in humans and various animal species.

Farm and companion animal organoids developed to date are summarized in Table 1. Early studies include the successful generation and transplantation of canine intestinal organoids (3) and the creation of chicken embryo-derived intestinal organoids (80). Since then, successful development of organoids and mechanistic studies utilizing organoids have been reported on a variety of tissues in various farm and companion animal species. At the same time, inter-species variations in optimum culture conditions have been noted in some tissues (44). Knowledge of such variations is important when considering translational applications of animal organoids. In the following subsections, we will provide the most recent updates on farm and companion animal organoids organized by organ systems and discuss their potential as One Health initiatives and in translational medicine, i.e., linking *in vitro* data to *in vivo* studies.

**Table 1:** Summary of normal, diseased, and applied organoid models described in selected farm and companion animal species to date.

**Animals**	**Models**				
	**Organs**	**Normal**	**Diseased**	**Applied**	**References**
**Farm animals**					
Pig	Esophagus	✓			(43)
	Intestine	✓			(44–48)
		✓	✓		(49)
		✓		✓	(23, 50–65)
			✓		(66, 67)
	Gallbladder	✓	✓		(68)
	Testis	✓			(69, 70)
Cattle	Intestine	✓			(10, 44, 71, 72)
		✓		✓	(60, 73, 74)
	Mammary gland	✓			(75)
	Oviduct	✓			(76)
Sheep	Intestine	✓			(44)
	Pancreas	✓		✓	(77)
Horse	Intestine	✓			(44, 78)
	Uterus	✓		✓	(79)
Chicken	Intestine	✓			(44, 80–83)
		✓		✓	(84, 85)
**Companion animals**					
Dog	Intestine	✓			(11, 44, 86)
		✓	✓		(87)
		✓		✓	(3)
	Liver		✓	✓	(88, 89)
	Kidney	✓			(90)
	Bladder		✓	✓	(91, 92)
	Prostate		✓	✓	(12)
	Skin	✓			(93, 94)
	Thyroid		✓		(95)
Cat	Intestine	✓			(44)
		✓		✓	(96)
	Liver	✓		✓	(4, 29)
Rabbit	Intestine	✓			(97)
		✓		✓	(98)

*Normal = Organoids derived from tissues/cells of normal animals*.

*Diseased = Organoids derived from tissues/cells of animals which developed spontaneous diseases*.

*Applied = Normal or disease model organoids that are used for applied research such as toxicity and efficacy testing of chemicals, drugs, and radiations, investigation of host-microbe interactions through bacterial co-culture, and validation studies as a source of cell transplantations and gene therapy*.

### Organoids of Digestive Systems

Farm and companion animal organoid models of digestive systems that have been described to date include those derived from tissues of esophagus, small intestine, and the colon. Successful development of intestinal organoids, referred to as enteroids or colonoids depending on the origin of the tissue, have been reported in various animal species.

In farm animals, porcine intestinal organoid model was first reported using distal duodenum and proximal jejunum of neonates (45). Since then porcine organoid models have been successfully developed from various sections of gastrointestinal tract (49, 50) such as esophageal submucosal gland (43), duodenum (23, 51–54), jejunum (23, 46, 51, 53–63, 66), ileum (23, 44, 47, 51, 53, 54, 64, 65), and the colon (48, 51, 53, 67). Several studies reported bovine organoids derived from jejunum (10, 60), ileum (44, 71, 73, 74), and the colon (72). Intestinal organoids of these species have been used in applied research such as investigation of epithelium-microbe interactions and modeling of bacterial, viral and parasitic infections (23, 51, 60, 74), some of which have important public health implications due to the risk of zoonotic infections. On the other hand, only a few studies can be found on equine and ovine organoids. Horse intestinal organoids were successfully cultured and expanded into 3D structure from jejunum (78) and ileum (44). Sheep intestinal organoids have been generated from ileum (44). Chicken intestinal organoids have been developed from small intestine (84), and more specifically from the sections of duodenum (85), jejunum (81), and cecum (44) in other studies. Furthermore, embryonic small intestines have been used to develop intestinal organoids in chickens (80, 82, 83, 85).

In companion animals, canine intestinal organoids have been successfully generated from duodenum, jejunum, ileum, and the colon using not only whole intestinal tissue sections but also much smaller endoscopic biopsy samples (3, 11, 44, 86, 87). The successful generation of disease model organoids has also been described using tissues derived from dogs with spontaneous gastrointestinal diseases such as inflammatory bowel disease and colorectal adenocarcinoma (87). Since these diseases have similar presentation and treatment options to those in humans, studies on canine enteroids and colonoids have potential application as a useful model for chronic gastrointestinal diseases (11). Cat ileum- and colon-derived organoids have been published with a potential to be used as a feline coronavirus infection model (44, 96). The establishment of rabbit intestinal organoid models have been reported using duodenal and cecal tissues (97, 98). These models provide a useful tool in biomedical research such as studies of human bacillary dysentery, which have been facing a major challenge due to a lack of representative animal models until recent development of infant rabbit infection models (99).

Conditioned medium containing “WRN factors” have been used as an effective growth medium in development and long-term maintenance of intestinal organoids in both humans and a variety of animals including pigs, cattle, sheep, horses, chickens, dogs, and rabbits (44, 100). The term WRN factors refers to Wnt3a, R-spondin and Noggin (44). Both Wnt3a and R-spondin are Wnt signaling pathway activators, which are essential for crypt proliferation, whereas Noggin is a bone morphogenetic protein (BMP) signaling pathway inhibitor which induces expansion of crypt numbers (101). Supplementation of these factors to the organoid culture media is considered critical in both human and animal organoids (100). However, relatively short-term maintenance, i.e., low passage number, of intestinal organoids was reported in cats (44). Optimization of culture conditions has been attempted to enhance productivity. For instance, addition of glycogen synthase kinase 3 (GSK-3) and rho-associated kinase (ROCK) inhibitors to the conditioned media containing WRN factors have been described in horses and dogs to improve intestinal stem cell survival (78, 87).

### Organoids of Hepatobiliary Systems

Besides in mice (102) and humans (103), the establishment of liver organoids has only been reported in dogs (88, 89) and cats (4, 29). A long-term hepatic organoid culture in companion animals was first reported in dogs using biopsy-derived tissue (88). Following this study, the establishment of a long-term feline hepatic organoid culture was described using ASCs derived from post-mortem tissue samples (4). In these studies, fresh or frozen liver tissues were cultured in Matrigel droplets and R-spondin-1-based culture medium, and they were successfully cryopreserved. It is important to note that the study demonstrated the importance of Wnt3a supplementation to the culture media to achieve long-term stable proliferation of canine hepatic organoids (88), This is in contrast to murine, human and feline hepatic organoids where the addition of Wnt3a is not needed for long-term culture (4, 102, 103).

Dogs with copper toxicity due to the COMMD-1 gene deficiency could provide the best preclinical model for investigation of inherited copper toxicosis in humans, such as Wilson's disease. Hepatic organoids from these dogs can be used to investigate the disease pathophysiology and new treatment strategies in copper toxicosis in both dogs and humans (88, 89). Canine liver organoids could also serve as a useful tool for investigation of infectious diseases such as viral hepatitis, which is also seen in humans (104). A greater predisposition for lipid accumulation in feline liver compared to humans would make feline hepatic organoids an excellent model for investigation of human lipid-storage diseases such as hepatic steatosis (4, 29).

Porcine gallbladder organoids are the only animal model that have been described concerning the biliary system (68). The study developed both normal and disease model organoids using tissues of gallbladder and cystic ducts and evaluated their functions such as anion and fluid secretions and mucus production. Since some pigs are born with naturally occurring cystic fibrosis, which results in a life-shortening multi-organ disorders including gallbladder due to genetic mutations and causes significant morbidity in humans, affected pigs present a good model for investigation of disease pathogenesis.

### Organoids of Pancreas

Sheep pancreatic duct organoids were generated using pancreatic duct cells from healthy fetus (77). The organoid was cultured in Matrigel containing EGF, R-Spondin-1, FGF10, and Noggin in Wnt3a-conditioned medium (77), which is a similar system described in mouse (105). Furthermore, this study demonstrated an importance of an appropriate amount of copper supplementation to the medium in order to achieve formation and growth of sheep pancreatic duct organoids (77). It is known that copper plays a pivotal role in maintaining normal physiology in mammalian cells, yet its optimal concentrations for tissue or organ development have not been determined. The finding in this study may provide a useful *in vitro* model to investigate the role of copper and its effective concentration in the context of organ development and physiology.

### Organoids of Urinary Systems

In a dog kidney organoid, budding tubule-like structures were formed from small pieces of renal tissues obtained from a euthanized dog (90). The study demonstrated that stem cell self-renewal and differentiation into tubular cells are promoted by high cell density, which induces signal transducer and activator of transcription-3 (STAT3) expression. Further, it was shown that treatment with either the STAT3 inhibitor AG490 or the STAT3 activator lipopolysaccharide reduced or increased colony forming efficiency in a dose-dependent manner, respectively. The findings would be useful for the development of normal human kidney stem cells considering their clinical applications and research such as investigations of nephrogenesis (106), nephrotoxicity screening (26), disease modeling, and regenerative medicine applications (107).

Bladder organoids were initially studied as an *ex vivo* model using mucosal biopsies obtained from porcine bladder (108). It was developed for preclinical experimental research such as pharmacology and toxicology screening, with the potential to replace animal models. More recently, neoplastic bladder organoid models have been described in dogs (91, 92). In these studies, bladder organoids were derived from the cells taken from urine samples. The disease model organoids were used to test effects of various anti-cancer drugs, demonstrating their use as a preclinical model for drug efficacy testing (91). Although normal bladder organoids have not been reported in animals other than mouse (109), such models in large animals would be useful for studies on normal physiology and host responses to external stimuli such as exposure to pathogens and antimicrobial drugs (110). Moreover, canine organoid models of bladder cancer would provide an important research tool for translational study including diagnostic biomarkers and optimization of therapeutic options as it resembles muscle-invasive bladder cancer in humans (91).

### Organoids of Male Reproductive Systems

Farm and companion animal organoid models that have been described to date concerning male reproductive systems include those of prostate and testis. For prostate, prostate cancer organoids have been reported using cancer cells in urine samples obtained from prostatic cancer bearing dogs (12). Similarly to bladder cancer organoids (91), the organoids were used to test effects of anti-cancer drugs and irradiation (12). Prostate cancer also occurs in humans and it bears poor prognosis in dogs (12). Therefore, the canine prostate cancer organoids would provide a useful translational model for advanced prostate cancer in humans, providing fresh insights into cancer treatment.

Testicular organoids have been successfully established from testicular tissues of pig, mouse, macaque (monkey), and human, where cells were harvested using a two-step enzymatic digestion process (69). Testis-specific cell types such as germ cells, Sertoli cells, Leydig cells, and peritubular myoid cells, were identified in these organoids. More recently, porcine organoids with vascular structures was reported for the first time allowing the coexistence of various cell types in organoid culture system (70).

In general, pigs are considered as superior animal models to mice due to their physiological and anatomical similarities to humans when considering biomedical and pharmaceutical studies (70). Therefore, studies on porcine testicular organoids possess wide application potentials for a variety of clinical and preclinical research including investigation of causes and treatment of male infertility and strategies to preserve fertility potentials in patients undertaking chemotherapy. Although testicular organoids have not been developed in other large animals, such studies would attract strong interests in the field of breeding industries which deal with high-value specimens such as horses and cattle (76). Moreover, farm animal organoid models provide useful tools to investigate reprotoxic effects of environmental stimuli or exogenous substances such as high ambient temperature, nutrition and water quality, and various chemicals such as antibiotics and food preservatives, which in turn would affect reproductive performance of economically important animals.

### Organoids of Female Reproductive Systems and Accessory Organs

Organoids of female reproductive systems that have been described to date concerning farm and companion animals are limited to those of endometrium in horses (79) and oviduct in cattle (76). The latter was only shown as a preliminary unpublished data (76). Equine endometrial organoids have been generated under similar conditions to those described for mice and humans (111, 112). However, the study demonstrated that R-spondin-1 and hepatocyte growth factor were not essential in growth of equine endometrial organoids, which was different from murine and human organoids. The horse endometrial organoids were established from both fresh and frozen-thawed endometrial biopsy samples. They retained the ability to maintain 3D structures and responded to hormonal stimuli, providing a novel *in vitro* culture model for evaluation of endometrial physiology, pathophysiology, and potential therapeutics for uterine diseases.

As for accessory organs of the female reproductive systems, development of mammary organoids have been reported using udder tissues in cattle (75). Another approach to establish a 3D culture model of primary bovine mammary epithelial cells has been described using cells obtained from milk (113). This promising model presents a great advantage of using non-invasively collected cells unlike the other studies. The technique will greatly contribute to preservation of animal welfare. Besides these studies, there is a single study which reported the development of a 3D culture model from caprine mammary gland tissue as a part of developing *in vitro* viral infection models (114).

Few studies have been reported on 3D culture of mammary tissue in pigs, although lactation of sows presents critical issue for survival of piglets. While further studies are needed to improve organoid technology in these species, the establishment of a robust technique for these species would provide a great insight into tissue biology such as development of mammary gland, mechanisms of lactation, and mechanisms of and host responses to bacterial and viral infections. Advances in the technology would not only contribute to welfare of these animals but also have economic impacts on farm animal industry by improving milk production.

In companion animals, dogs has been suggested as an excellent animal model for human breast cancer due to many clinical and molecular similarities such as spontaneous development of the disease with an intact immune system, age of onset, hormonal etiology, disease progression and outcomes and gene expressions (115). Organoids of spontaneously occurring canine mammary tumors could present great potential for cancer research, drug development, and treatment trials by serving as a good preclinical model.

### Organoids of Integumentary System

Studies to develop 3D skin models in animals originally started for use in the pharmaceutical and cosmetic industries (116). Initially, pig skin was considered a good model to test drugs of topical formulation (117). At present, availability of animal skin models are very limited due to the development of human 3D skin models and most of them are developed for use in veterinary medicine or comparative biology research (116). Nonetheless, investigation of animal skin models possesses some values in studies on comparative biology and skin diseases such as bacterial and atopic dermatitis in dogs, which hold similar clinical course and presentation to those in humans (118). Additionally, skin models of farm animals, especially those of which skin components may be included for human consumption such as poultry, may serve as a good tool to evaluate impacts of toxic substances on skin.

The studies on canine keratinocyte and epidermal organoid culture systems have been reported (93, 94). In the study, keratinocyte organoids from both microdissected interfollicular epidermis and hair follicle tissues were successfully established using culture medium containing Noggin, R-spondin-1, and Rho kinase inhibitor (93). The authors reported that hair follicle-derived organoids grew best when the medium was supplemented with fibroblast growth factor (FGF) 2, and FGF10, whereas interfollicular epidermis derived organoids grew best when the medium was supplemented with epidermal growth factor (EGF), FGF10, Forskolin and transforming growth factor (TGF) β inhibitor. The study also demonstrated that Wnt3a was not essential to grow canine keratinocyte organoids, thus was removed from the expansion media. Furthermore, an attempt to optimize the canine keratinocyte organoid system and develop an epidermal organoid revealed that supplementation of Wnt3a and other factors to differentiation medium did not have beneficial effects on inducing differentiation of the interfollicular epidermis-derived organoids closer to normal epidermis (94).

### Organoids of Endocrine Systems

Thyroid organoids have been successfully generated from canine follicular cell thyroid carcinomas (FTCs) (95). Canine FTC-derived organoids conserved the expression of proteins involved in iodine uptake, and hence opened new research possibilities as an *in vitro* model to modulate iodine uptake and improve radioiodine therapy for thyroid cancer. While development of thyroid cancer following transplantation of genetically engineered oncogenic thyroid organoids has been described in mouse (119), this is the first organoid model which is derived from spontaneous thyroid cancer in any species (95). Since FTCs, although rare, do occurs in humans (120), canine spontaneous cancer organoids would serve as a useful model for preclinical studies on human FTCs.

## Roles Of Farm and Companion Animal Organoids In One Health Initiative

### Public Health: Farm Animal Organoids as a Tool for Studying Zoonotic Infections

There are many shared health threats between humans and animals. They include not only zoonotic infectious diseases but also non-infectious diseases such as metabolic disorders, chronic inflammatory disorders, and neoplastic diseases, antibiotic resistance, and environmental contamination such as toxic agents in the air, soil and drinking water. Among these, zoonotic infectious diseases are particularly important in the context of public health.

With growing global human populations and advancement in transportation systems, points of contact between humans and animals increase (18). It is now known that many animals including both wildlife and domestic species play an important role in the spread of zoonotic diseases as potential reservoirs. For instance, severe acute respiratory syndrome (SARS), which resulted in a major outbreak worldwide in early 2000s, and the current ongoing pandemic of the new coronavirus 2019 disease (COVID-19) are caused by previously unrecognized coronaviruses which are most likely evolved from bats as a natural reservoir (SARS-CoV and SARS-CoV-2, respectively) (18, 121). A recent study demonstrated susceptibility of bat intestinal organoids to SARS-CoV-2 and sustained viral replications in organoids, which could not have been achieved using bat cell lines, by establishing an *in vitro* infection model (122). This study demonstrated previously unculturable novel viruses in established cell lines could be cultured in organoids. Additionally, SARS-CoV-2 has been shown to infect various domestic and wildlife species other than humans including dogs, cats, minks, ferrets, hamsters, lions and tigers (121). Animal organoids derived from different species would provide an effective tool to evaluate cross-species variations in viral susceptibility or species-specific infectivity of viruses with zoonotic and zooanthroponotic potentials, providing a great alternative to animal models for infection studies.

Besides wildlife, domestic animal species also play an important role in public health. Some pathogens that infect livestock, causing clinical or subclinical diseases, also infect humans and cause clinical diseases in susceptible individuals or in a group of people, leading to epidemics, e.g., *Salmonella typhimurium, Escherichia coli, Toxoplasma gondii*, and *Giardia duodenalis*. To investigate host-pathogen interactions and disease responses upon exposure to potential pathogens, infectious disease models have been created by co-culturing healthy intestinal organoids from livestock with pathogens or bacterial toxins. The reported models include those infected with *Salmonella typhimurium* and *Toxoplasma gondii* in pigs and cattle (60) and Enterotoxigenic *Escherichia coli* (ETEC) in pigs (23), and those exposed to Shiga toxins produced by Enterohemorrhagic *Escherichia coli* (EHEC) in cattle (73). These models offered a useful alternative to animal models, which require a large amount of resources such as money, labor and housing facilities. Besides enabling significant cost reduction, they provided a new insight for mechanisms of disease development and effects of virulence factors on host epithelium through creating a model closely mimicking the *in vivo* tissue. Also, since many pathogens have host specificity, farm animal organoids could serve as a good model to study host-pathogen interactions and protective mechanisms of hosts when organoids of asymptomatic carrier species are used. Furthermore, infection models could serve as useful tools for screening efficacy and adverse events of vaccines and antibiotics against infectious diseases, thus helping to improve public health.

### Food Security: Farm Animal Organoids as a Tool for Improving Agricultural Production

Humans are always in danger of falling into a severe food shortage due to the increase in the world population, climate change caused by global warming, and the spread of infectious diseases (20, 123). It has been proposed that more than 50% increase in food production compared to the production levels in 2012 will be necessary to fulfill the needs of global population by 2050 (20).

To date, organoids of digestive systems have been developed in multiple farm animal species. They have been used for studies concerning nutrition, toxins and infectious diseases with a great expectation to improve productivity in one way or another. For example, porcine intestinal organoids have been used to evaluate effects of different nutrients such as dietary fiber (65), glutamate (59), and vitamin A (63) as well as fungal-derived toxins such as Deoxynivalenol (DON) (55) on development and homeostasis of intestinal epithelial cells. Organoids of ovine pancreatic duct have been used to investigate the effect of dietary copper on organ growth (77). Organoids of chicken intestine have been used to assess how different chemicals such as growth factors, hormones, vitamins, pesticides, enterotoxins, endotoxins, mycotoxins and *Lactobacillus acidophilus* bacteria affect the cells (84, 85). All these studies have important implications for improving feed efficiency by providing potentially useful information to formulate a superior dietary composition to achieve enhanced gut health and tissue development, hence helping to achieve improved animal growth.

Moreover, studies on infectious diseases using organoids would not only contribute to improving production efficiency of farm animals through decreased morbidity and mortality but also minimize economical damage for infectious disease management. In addition to the bacterial and parasitic infection models described in the previous section, several viral infection models have been established using porcine intestinal organoids. They include infections with enteric coronavirus (ECoV) such as porcine epidemic diarrhea virus (PEDV) (50, 51), porcine deltacoronavirus (PDCoV) (53), and transmissible gastroenteritis virus (TGEV) (62). Although these viruses have significant economic impact in the pig industry due to high morbidity and mortality in piglets (124), the lack of effective *in vitro* models has been a limiting factor for in depth studies on these diseases. This was mainly due to poor infectivity and replicability of viruses in *in vitro* culture systems (62). However, with the emergence of organoid technology and with the development of effective *in vitro* infection models, it is expected that these models would greatly help scientists to understand pathogenesis of these important diseases. Knowledge obtained through organoid studies would offer new insights to improve herd health management strategies in the long run, contributing to the reduction of economic losses as well as the increase in agricultural production.

Although organoids of reproductive systems and applications thereof in farm animals remain very limited, their potential for improving reproductive success are unlimited as discussed in the earlier sections. Improved milk production would not only have great economic benefit to dairy industry, but also to other industries dealing with food production animals as both quality and quantity of lactation determine survival of offspring, which is critical for sustainability of the industry. Similarly, studies relating to male and female fertility and maintenance of pregnancy to term also comprise important aspects of reproductive success. Therefore, further studies must be conducted to develop robust organoid culture techniques in these tissues in major food production animals.

### Comparative Medicine: Companion Animal Organoids as a Tool for Improving Human Health

Organoids derived from human patients would be a superior tool to those of animal counterparts when applications for personalized medicine such as patient-specific drug efficacy testing and cell transplantation to individual patient are set as the primary goal of organoid studies (125). However, there will still be a gap in translatability between *in vitro* findings and *in vivo* application even using human samples. Companion animals which develop diseases similar to those in humans can play a pivotal role in fundamental studies on pathophysiology or preclinical studies on safety and efficacy of new therapeutics (16). There would be a value in investigating cross-species similarities and differences in drug safety and efficacy to better predict their performance in human clinical trials as the Food and Drug Administration (FDA) requires preclinical safety and efficacy studies from various animal species in addition to mice prior to human trials (126). Therefore, using organoids from companion animals with naturally occurring diseases can not only model diseases of humans *in vitro* but select the most promising drug candidates for such diseases. Subsequently, those drug candidates can be tested in companion animals with the disease prior to human clinical trials. This pipeline of preclinical studies using animal patients which suffer from naturally occurring diseases will also allow such veterinary patients to receive benefits of new potential therapeutics while they provide the data which bridge between preclinical and human clinical trials. Moreover, studies using organoids from various animal species (i.e., cross-species examination) would contribute to reducing failure rates in human clinical study, thus accelerating approval of new drugs. This approach could also contribute to achieving strong ethical benefits according to 3R principle (reduce, refine, replace) by avoiding, or reducing if not complete, the use of laboratory animals for the purpose of *in vivo* preclinical trials in new drug development.

A large number of case-derived tissue samples are necessary to create various types of disease model organoids and use them for research with reasonable reliability. With the recent increase in the number of companion animals who receive life-long veterinary care, reports on animal diseases which have similarities to those seen in humans have been growing (16, 127). Therefore, strategic collaboration with veterinary medicine would accelerate accumulation of disease samples which are common between humans and animals, helping to develop a solid base, or so called disease organoid biobanks, for translational research.

## Evolution Of Organoid Culture Systems

**Figure 1:**
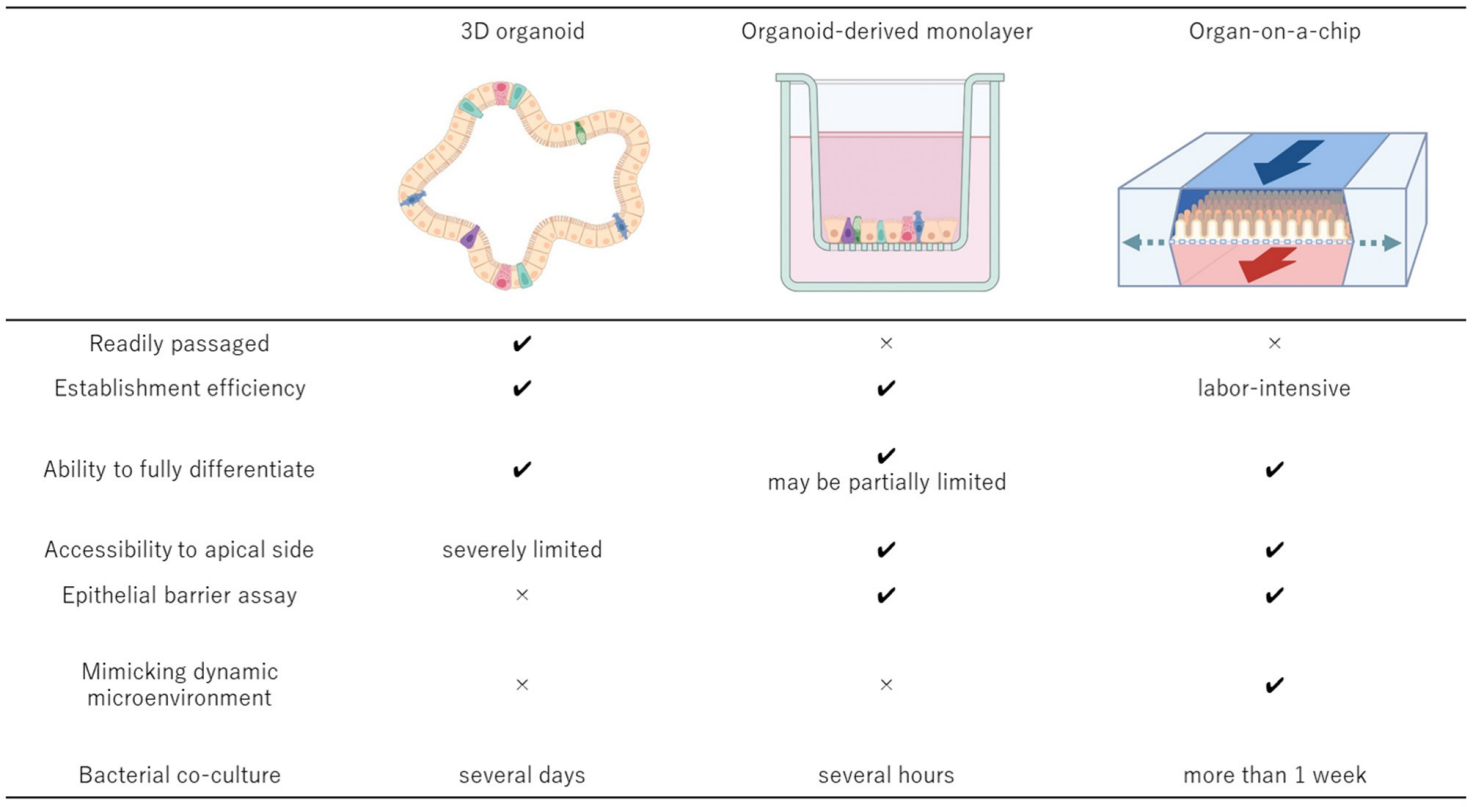
Comparisons of the key features of the 3D organoid culture system, organoid-derived 2D monolayer culture system, and organ-on-a-chip system. The 3D organoids should be derived from the relevant donors to develop the cell culture system that can be established efficiently and passaged readily while maintaining the physiologic cell diversity. To accomplish more advanced epithelial interface investigation, organoid-derived 2D monolayer can be established using conventional Transwell system or other well established 2D culture systems. Integration of the 3D organoid culture system and organ-on-a-chip technologies provides the most suitable system to investigate more complex microenvironment with host cells and microbial cells because of its dynamic environment with mechanical motions and the ability to manipulate the oxygen gradient within the system. Created with BioRender.com.

Farm and companion animal organoids have a great potential for contributing to human health based on One Health initiatives. Organoids would continue to strengthen their presence in the fields of biology and life science as the technology becomes more robust and efficient in the next decades. The 3D organoid culture system is a relatively new technique and possesses unique characteristics from those of traditional 2D cell culture system. Other advanced culture systems such as organoid-derived 2D monolayer and organ-on-a-chip systems have been emerging to overcome the weakness in current organoid systems (Figure 1). These advanced culture platforms offer great potential to widen the scope of research through dissociation of 3D organoids into single cells and their integration into the advanced culture systems.

### Traditional 2D Cell Culture System

2D cell culture systems including primary cells and cell lines have been widely used as *in vitro* models since the early 1900s (128). Primary cell culture uses the cells which are freshly isolated from a donor. Although these cells are considered to behave similarly to those in the living organism due to the fact that the cells are cultured shortly after they are harvested from a donor, they have limited proliferation potential and tend to lose their phenotypic characteristics over time (26). On the other hand, immortalized cell lines can be stably cultured for long time and have homogenous traits. Cell lines are usually derived from tumor tissues or created via *in vitro* viral transformation to become artificially cancerous (2). Although these cell lines can grow and divide continuously, tumor-derived cell lines usually have chromosomal mutations which affect cellular metabolism and physiology (129, 130). Such abnormalities also evolve during passage, leading to problems with reproducibility (130). Additionally, immortalized cell lines may not fully represent normal traits of the original tissue because the process of immortalization, or changes in genomic contents, may alter cellular characteristics and functions (26). Hence, an alternative *in vitro* model that can sustain multiple passages over prolonged periods while maintaining normal cellular functions and characteristics of the original tissue and possibly a living organism has been sought after.

### 3D Organoid Culture System

3D organoid culture system is a newly developed technique which provides more physiologic cell populations and microenvironment to perform biomedical research. 3D organoid culture relies on proliferation and differentiation of stem cells (131); therefore, organoids retain “stemness” as the original organ (2). They contain multiple cell types which collectively exhibit organ-like phenotypes, architectures, and functions including cell-cell interactions similar to those observed in the original tissue (131). For instance, intestinal organoids have multicellular composition with intestinal stem cells, enterocytes, Goblet cells, Paneth cells, and enteroendocrine cells (101), expressing polarization as an intestinal epithelium (5). These cells are well differentiated and can reproduce the fine structures of intestinal lumen consisting of crypts and villi (5, 42) and cell junctions, which are common to *in vivo* tissues, enabling effective cell-cell communication through the exchange of ions, secretion of vesicles, and transduction of electrical stimulations (128, 132). Moreover, these organoids show the biological functions of gut, such as absorption and secretion (5, 42). These features make organoids very useful for specialized experiments such as swelling assays that simulate fluid secretion in the body (133).

Another benefit of 3D organoid culture is the fact that they can be isolated from both normal and diseased donors. Since organoid cultures maintain the traits of the original tissue, organoids derived from tissues of clinical cases can recapitulate the pathology of disease (133–136). Therefore, patient-derived organoids are very useful natural models for studying pathophysiology of the disease (135, 136) and establishing disease-specific living biobanks for large scale genetic analyses such as in cases of neoplasia (137–140). Patient-derived organoid models also present great potential for precision medicine such as preclinical drug efficacy testing and pharmacodynamics studies (141, 142).

The 3D organoid culture can be expanded for long time on the scale of months or even 1 year or longer, which has been reported in multiple studies involving various tissues such as gastrointestinal tract, liver and pancreas taken from both humans and animals (42, 44, 101–103, 105, 143). Additionally, organoids can be cryopreserved and re-cultured from cryopreserved samples, maintaining cellular characteristics similar to that of the original organoids (144). Successful recovery of cryopreserved organoids (44, 145) can provide a banked resource for future use. Furthermore, organoids are useful for standard biological analyses such as live cell imaging using inverted phase-contrast and confocal microscopies (101), gene expression analysis (14), genetic modification (146), and enzyme activity assay (14).

Although organoids are a promising model with many advantages as described above, their self-organizing capacity could become a limitation when studies that necessitate apical access to the cells are considered. 3D organoids form hollow cystic structures as they grow up in extracellular matrix such as Matrigel (147, 148). This structure makes it very difficult not only to study interactions between epithelial cells and exogenous substances such as drugs, nutrients, toxins and microbial pathogens, but also to investigate functions and cell-cell interactions of epithelium such as intracellular transport, secretion, and absorption. To overcome this limitation, microinjection technique has been used to introduce enteric pathogens or other compounds into organoid lumen (149, 150). However, microinjection is labor-intensive and has low success rates without special equipment and experienced technicians (151), making the technique unsuitable for high-throughput applications. Moreover, an invasive process of this technique has a possibility to compromise the epithelial barrier function, resulting in unintended leakage.

### Organoid-Derived 2D Monolayer Culture System

More recently, organoid-derived 2D monolayer culture models have become increasingly described in multiple animal species including pigs (23, 46, 47, 49–51, 53, 54, 58, 62, 64), rabbits (97, 98), cattle (72), and dogs (11). This culture system was developed to get over the limitation of 3D culture system which arose when intestinal organoids are to be used in studies. The major advantage of the organoid-derived 2D monolayer system is that it provides much easier access to the luminal surface compared to the conventional 3D organoid culture, and thus serving as a better *in vitro* model to study epithelial interaction to luminal microenvironment for preclinical drug testing and infection modeling (23, 50, 51, 53, 54, 62, 64, 98). Comparing to the traditional 2D cell culture system which includes only one cell type, the organoid-derived nature of this system provides a physiologically more representative model to *in vivo* tissue due to greater cellular diversity. Additionally, by culturing the cells on a Transwell insert, basolateral access to the cells in addition to normal apical access becomes feasible, enabling studies involving transport assays.

In this system, intestinal organoids are enzymatically dissociated into single cells, then seeded on top of nanoporous Transwell inserts pre-coated with Matrigel and collagen (11). Unlike other technique involving physical disruption of 3D organoids into fragments, which is used in a study to establish enteric infection models (60), cell polarization is preserved and accessibility to the apical side is much improved, making this model better suited for studies to investigate epithelial-luminal interactions with a high-throughput procedure. Furthermore, this model can also be used to evaluate integrity of epithelial tight junction barriers by measuring transepithelial electrical resistance (TEER) and apparent paracellular permeability (Papp) values (11).

However, the organoid-derived 2D monolayer system also has drawbacks in comparison to the 3D organoid system. For example, the Paneth cell marker lysozyme positive cells that were present in the original murine 3D organoids were not detected in the 2D monolayer culture (152). Although this was not the case when porcine intestinal organoids were dissociated and seeded to 2D monolayer culture (47), the result raises a concern as to whether this 2D monolayer culture system could reproduce the pathophysiological features of the *in vivo* intestine due to the lack of an important cell type. Moreover, organoid-derived 2D monolayer cultures cannot be easily passaged and propagated (153) as opposed to the 3D organoid cultures. In addition, 2D monolayer cultures on Transwell inserts cannot be co-cultured with bacteria for a long time as microbial overgrowth tends to occur under static culture conditions due to accumulation of the waste products and consumption of nutrients (100, 154). Therefore, host-pathogen interactions can only be evaluated for a short period of time, usually from half an hour to several hours (154). Since crosstalk between epithelial cells and commensal bacterial flora occurs constantly in tissues *in vivo*, it is important to establish a stable co-culture system that can be maintained for a prolonged period in order to improve our understanding of host-microbe interactions (155).

### Microfluidic Organ-on-a-Chip Technology

While 3D organoid and organoid-derived 2D monolayer cultures are powerful tools for both fundamental and applied research, it has been a big challenge to develop more complex systems which better represent dynamic tissue-tissue interactions occurring *in vivo* organs. A major difference from the 3D organoid and organoid-derived 2D monolayer cultures is that the organ-on-a-chip system cultures cells in a continuously perfused chamber to remove the waste product and supply continuous nutrients. It allows *in vitro* organ models to recapitulate mechanical movement of tissue, air, and fluid and their interactions within a defined culture system which contains minimum functional units of an organ (156). The system also allows stable bacterial co-culture for a relatively long period without causing bacterial overgrowth (155, 157), unlike the other static culture systems. This is crucial benefit that microfluidic technology can offer especially when investigating host-microbe interactions that occur in healthy tissue as well as in the face of challenge with bacterial pathogens. Greater control over various parameters within the culture system compared with the other static cultures facilitates more advanced studies dealing with various physiological functions and responses to external stimuli.

Various organ-on-a-chip systems consist of two closely apposed microchannels which are separated by a thin, porous membrane. While the system allows culture conditions of the two channels to be controlled separately from each other, cells on each side are capable of communicating with each other through the microporous membrane (158). This unique structure of the system has made it possible to create more relevant organ-specific microenvironment *in vitro*. Some of microfluidic devices allow the application of vacuum to the chambers and create pressure-driven deformation to the chamber wall, imitating dynamic stretching associated with breathing or peristaltic movement of the gut. In a gut-on-a-chip system, vascular endothelial cells and intestinal epithelial cells are cultured on each side to create lumen-capillary interface. Dynamic condition mimicking the *in vivo* intestine is created through introduction of luminal flow with varying nutrient gradients and bacterial contents, inflammatory cytokines as well as peristalsis-like mechanical movement (157). Anoxic-oxic interface can also be created by flowing culture medium with controlled oxygen gradient into each compartment (155). The system has been applied to investigate effects of microbiome, inflammatory cells, and mechanical movement on intestinal health and cellular differentiation under physiologically more relevant conditions (157, 159).

To date, several different organ-on-a-chip devices have been successfully developed in humans, which include, liver (160), brain (161, 162), heart (163, 164), kidney (165), and retina (166, 167) besides lung (158) and intestine (168). They have been used in studies on physiology (167), pathology (169), toxicology (161, 165, 170), and pharmacology (164, 171–173). On the other hand, there are only two reports regarding application of the organ-on-a-chip technology to farm and companion animals, which described canine and bovine oviduct-on-a-chip systems, respectively (174, 175). Each of these studies described potential of animal derived-organ-on-a-chip models as a disease model relevant to humans and *in vitro* fertilization studies. Organ-on-a-chip systems derived from farm and companion animals would contribute to fill the gap that organoid cultures and static monolayer systems could not support adequately. Further advances in the organ-on-a-chip technology and its application would greatly improve our understanding of fundamental biology and pathology, thus enhancing health care management of both animals and humans.

## Conclusions and Future Perspective

**Figure 2:**
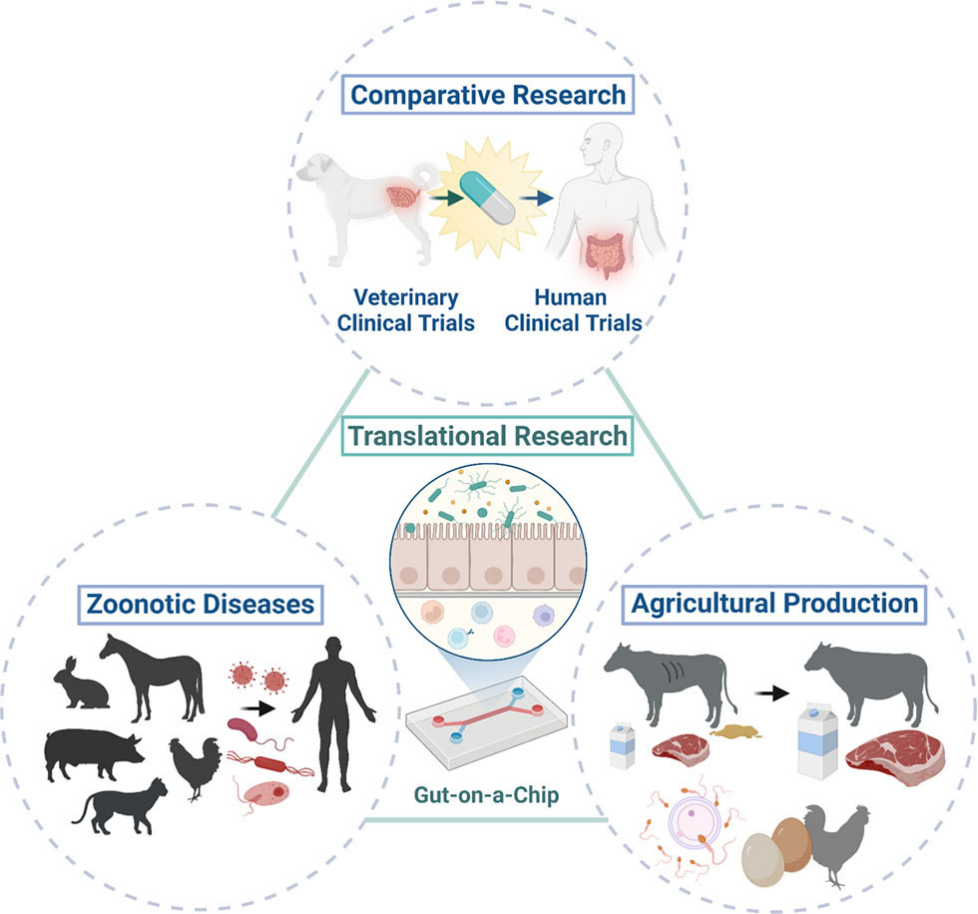
One Health initiatives with the integration of animal organoids and organ-on-a-chip technologies. Integration of animal organoids and organ-on-a-chip technologies will allow translational research (i.e., bench to bedside research) in various animal species and humans to enable cross-species investigation of physiology in health and disease. This allows the application of these technologies to comparative research and potentially efficient drug discovery with the use of natural animal disease models because of the similar environment, diet, and disease development that they share with humans. Investigations of host-pathogen interactions in zoonotic infectious diseases using animal organoids can improve public health through translational research as some animals only develop mild clinical diseases or serve as asymptomatic carriers upon exposure to potential pathogens which can cause severe clinical diseases in humans. Further mechanistic and novel therapeutic investigations in various pathogenic and wasting diseases (i.e., enteric pathogens) or reproductive diseases can be performed with translational medicine using farm animal organoids, which could ultimately contribute to improve the agricultural production to meet ever-growing human needs. Created with BioRender.com.

Animal organoids from farm and companion animals have made and can make significant contributions to human health as One Health initiatives (Figure 2). Farm and companion animal organoid models can offer a useful tool to investigate host-pathogen interactions and host defense mechanisms against zoonotic infectious diseases efficiently especially when the species are affected only mildly or sub-clinically. Other infectious or non-infectious disease organoid models of farm animals would provide new insights for improving heard health and agricultural productivity through improved disease management and reproductive success, leading to sustainable food production. Furthermore, disease organoids from companion animals affected with naturally occurring diseases can not only serve as a useful model for human diseases but also provide a good candidate for preclinical drug screening for the development of effective treatment.

There are various new culture platforms established to further strengthen what organoids can offer in biomedical research including organoid-derived 2D monolayer and organ-on-a-chip technology. Improvement in the complexity of the experimental designs can provide an essential *in vitro* platform to support the 3R principles (reduce, refine, replace the use of laboratory animals in new drug development) and contribute to the health and welfare of animals as well as to enhance the health of humans through translational research. Finally, we envision that the establishment of organ models integrating 3D organoid culture and organ-on-a-chip systems could lead to deeper insights in a range of different pathological conditions and broaden opportunities as One Health initiatives.

## Author Contributions

MK, TG, YT, and IN wrote the original manuscript. MK and YA edited the manuscript. MK, TG, YT, and YA designed and wrote the tables and figures. All authors contributed to the article and approved the submitted version.

## Funding

This work was supported in part by the Office of the Director, National Institutes of Health (K01OD030515 and R21OD031903 to YA).

## Conflict of Interest

The authors declare that the research was conducted in the absence of any commercial or financial relationships that could be construed as a potential conflict of interest. The handling editor WS declared a past co-authorship with the author YA.

## Publisher's Note

All claims expressed in this article are solely those of the authors and do not necessarily represent those of their affiliated organizations, or those of the publisher, the editors and the reviewers. Any product that may be evaluated in this article, or claim that may be made by its manufacturer, is not guaranteed or endorsed by the publisher.

## References

[1] Seeger B. Farm animal-derived models of the intestinal epithelium: recent advances and future applications of intestinal organoids. Altern Lab Anim. (2020) 48:215–33. 10.1177/0261192920974026 33337913

[2] Kar SK Wells JM Ellen ED te Pas MFW Madsen O Groenen MAM. Organoids: a promising new *in vitro* platform in livestock and veterinary research. Vet Res. (2021) 52:43. 10.1186/s13567-021-00904-2 33691792 PMC7943711

[3] Agopian VG Chen DC Avansino JR Stelzner M. Intestinal stem cell organoid transplantation generates neomucosa in dogs. J Gastrointest Surg. (2009) 13:971–82. 10.1007/s11605-009-0806-x 19165549

[4] Kruitwagen HS Oosterhoff LA Vernooij IGWH Schrall IM van Wolferen ME Bannink F. Long-term adult feline liver organoid cultures for disease modeling of hepatic steatosis. Stem Cell Reports. (2017) 8:822–30. 10.1016/j.stemcr.2017.02.015 28344000 PMC5390114

[5] Spence JR Mayhew CN Rankin SA Kuhar MF Vallance JE Tolle K. Directed differentiation of human pluripotent stem cells into intestinal tissue *in vitro*. Nature. (2011) 470:105–10. 10.1038/nature09691 21151107 PMC3033971

[6] Matsui TK Matsubayashi M Sakaguchi YM Hayashi RK Zheng C Sugie K. Six-month cultured cerebral organoids from human ES cells contain matured neural cells. Neurosci Lett. (2018) 670:75–82. 10.1016/j.neulet.2018.01.040 29398520

[7] Bershteyn M Nowakowski TJ Pollen AA Di Lullo E Nene A Wynshaw-Boris A. Human iPSC-derived cerebral organoids model cellular features of lissencephaly and reveal prolonged mitosis of outer radial glia. Cell Stem Cell. (2017) 20:435–49.e4. 10.1016/j.stem.2016.12.007 28111201 PMC5667944

[8] Akbari S Sevinç GG Ersoy N Basak O Kaplan K Sevinç K. Robust, long-term culture of endoderm-derived hepatic organoids for disease modeling. Stem Cell Reports. (2019) 13:627–41. 10.1016/j.stemcr.2019.08.007 31522975 PMC6829764

[9] Prochazkova M Chavez MG Prochazka J Felfy H Mushegyan V Klein OD. Embryonic versus adult stem cells. In: Vishwakarma A, Sarpe P, Shi S, Ramalingam M, editors. Stem Cell Biology and Tissue Engineering in Dental Sciences. New York, NY: Elsevier Inc (2015). p. 249–62.

[10] Lee BR Yang H Lee SI Haq I Ock SA Wi H. Robust three-dimensional (3d) expansion of bovine intestinal organoids: an *in vitro* model as a potential alternative to an *in vivo* system. Animals. (2021) 11:2115. 10.21203/rs.3.rs-164747/v1 34359243 PMC8300217

[11] Ambrosini YM Park Y Jergens AE Shin W Min S Atherly T. Recapitulation of the accessible interface of biopsy-derived canine intestinal organoids to study epithelial-luminal interactions. PLoS ONE. (2020) 15:e0231423. 10.1371/journal.pone.0231423 32302323 PMC7164685

[12] Usui T Sakurai M Nishikawa S Umata K Nemoto Y Haraguchi T. Establishment of a dog primary prostate cancer organoid using the urine cancer stem cells. Cancer Sci. (2017) 108:2383–92. 10.1111/cas.13418 29024204 PMC5715251

[13] Takashima GK Day MJ. Setting the one health Agenda and the human-companion animal bond. Int J Environ Res Public Health. (2014) 11:11110–20. 10.3390/ijerph111111110 25350006 PMC4245602

[14] Janssen AWF Duivenvoorde LPM Rijkers D Nijssen R Peijnenburg AACM van der Zande M. Cytochrome P450 expression, induction and activity in human induced pluripotent stem cell-derived intestinal organoids and comparison with primary human intestinal epithelial cells and Caco-2 cells. Arch Toxicol. (2020) 95:907–22. 10.1007/s00204-020-02953-6 33263786 PMC7904554

[15] Lee J B?scke R Tang PC Hartman BH Heller S Koehler KR. Hair follicle development in mouse pluripotent stem cell-derived skin organoids. Cell Rep. (2018) 22:242–54. 10.1016/j.celrep.2017.12.007 29298425 PMC5806130

[16] Kol A Arzi B Athanasiou KA Farmer DL Nolta JA Rebhun RB. Companion animals: translational scientist's new best friends. Sci Transl Med. (2015) 7:aaa9116. 10.1126/scitranslmed.aaa9116 26446953 PMC4806851

[17] Alvarez CE. Naturally occurring cancers in dogs: insights for translational genetics and medicine. ILAR J. (2014) 55:16–45. 10.1093/ilar/ilu010 24936028

[18] Mackenzie JS McKinnon M Jeggo M. One health: from concept to practice. In: Yamada A, Kahn L, Kaplan B, Monath T, Woodall J, Conti L, editors. Confronting Emerging Zoonoses. Tokyo: Springer (2014). 10.1007/978-4-431-55120-1_8

[19] Lerner H Berg C. The concept of health in One Health and some practical implications for research and education: what is One Health? Infect Ecol Epidemiol. (2015) 5:25300. 10.3402/iee.v5.25300 25660757 PMC4320999

[20] Garcia SN Osburn BI Jay-Russell MT. One health for food safety, food security, and sustainable food production. Front Sustain Food Syst. (2020) 4:e0001. 10.3389/fsufs.2020.00001

[21] Lei M Schumacher LJ Lai YC Juan WT Yeh CY Wu P. Self-organization process in newborn skin organoid formation inspires strategy to restore hair regeneration of adult cells. Proc Natl Acad Sci USA. (2017) 114:E7101–10. 10.1073/pnas.1700475114 28798065 PMC5576784

[22] Sakib S Goldsmith T Voigt A Dobrinski I. Testicular organoids to study cell–cell interactions in the mammalian testis. Andrology. (2020) 8:835–41. 10.1111/andr.12680 31328437 PMC7786437

[23] Vermeire B Gonzalez LM Jansens RJJ Cox E Devriendt B. Porcine small intestinal organoids as a model to explore ETEC-host interactions in the gut. Vet Res. (2021) 52:94. 10.1186/s13567-021-00961-7 34174960 PMC8235647

[24] Yin Y Bijvelds M Dang W Xu L Van Der Eijk AA Knipping K. Modeling rotavirus infection and antiviral therapy using primary intestinal organoids. Antiviral Res. (2015) 123:120–31. 10.1016/j.antiviral.2015.09.010 26408355

[25] Yin Y Zhou D. Organoid and enteroid modeling of Salmonella Infection. Front Cell Infect Microbiol. (2018) 8:e00102. 10.3389/fcimb.2018.00102 29670862 PMC5894114

[26] Faria J Ahmed S Gerritsen KGF Mihaila SM Masereeuw R. Kidney-based *in vitro* models for drug-induced toxicity testing. Arch Toxicol. (2019) 93:3397–418. 10.1007/s00204-019-02598-0 31664498

[27] Augustyniak J Bertero A Coccini T Baderna D Buzanska L Caloni F. Organoids are promising tools for species-specific *in vitro* toxicological studies. J Appl Toxicol. (2019) 39:1610–22. 10.1002/jat.3815 31168795

[28] Duarte AA Gogola E Sachs N Barazas M Annunziato S De Ruiter JR. BRCA-deficient mouse mammary tumor organoids to study cancer-drug resistance. Nat Methods. (2018) 15:134–40. 10.1038/nmeth.4535 29256493

[29] Haaker MW Kruitwagen HS Vaandrager AB Houweling M Penning LC Molenaar MR. Identification of potential drugs for treatment of hepatic lipidosis in cats using an *in vitro* feline liver organoid system. J Vet Intern Med. (2020) 34:132–8. 10.1111/jvim.15670 31830357 PMC6979087

[30] Langhans SA. Three-dimensional *in vitro* cell culture models in drug discovery and drug repositioning. Front Pharmacol. (2018) 9:6. 10.3389/fphar.2018.00006 29410625 PMC5787088

[31] Homicsko K. Organoid technology and applications in cancer immunotherapy and precision medicine. Curr Opin Biotechnol. (2020) 65:242–7. 10.1016/j.copbio.2020.05.002 32603978

[32] Wilson HV. A new method by which sponges may be artificially reared. Science. (1907) 25:912–5. 10.1126/science.25.649.912 17842577

[33] Holtfreter J. Experimental studies on the development of the pronephros. Rev Can Biol. (1943) 3:220–50.

[34] Weiss P Taylor AC. Reconstitution of complete organs from single-cell suspensions of chick embryos in advanced stages of differentiation. Proc Natl Acad Sci. (1960) 46:1177–85. 10.1073/pnas.46.9.1177 16590731 PMC223021

[35] Evans M. Origin of mouse embryonal carcinoma cells and the possibility of their direct isolation into tissue culture. J Reprod Fertil. (1981) 62:625–31. 10.1530/jrf.0.0620625 7019433

[36] Martin GR. Isolation of a pluripotent cell line from early mouse embryos cultured in medium conditioned by teratocarcinoma stem cells. Proc Natl Acad Sci USA. (1981) 78:7634–8. 10.1073/pnas.78.12.7634 6950406 PMC349323

[37] Thomson JA Itskovitz-Eldor J Shapiro SS Waknitz MA Swiergiel JJ Marshall VS. Embryonic stem cell lines derived from human blastocysts. Science. (1998) 282:1145–7. 10.1126/science.282.5391.1145 9804556

[38] Takahashi K Yamanaka S. Induction of pluripotent stem cells from mouse embryonic and adult fibroblast cultures by defined factors. Cell. (2006) 126:663–76. 10.1016/j.cell.2006.07.024 16904174

[39] Takahashi K Tanabe K Ohnuki M Narita M Ichisaka T Tomoda K. Induction of pluripotent stem cells from adult human fibroblasts by defined factors. Cell. (2007) 131:861–72. 10.1016/j.cell.2007.11.019 18035408

[40] Yu J Vodyanik MA Smuga-Otto K Antosiewicz-Bourget J Frane JL Tian S. Induced pluripotent stem cell lines derived from human somatic cells. Science. (2007) 318:1917–20. 10.1126/science.1151526 18029452

[41] Eiraku M Watanabe K Matsuo-Takasaki M Kawada M Yonemura S Matsumura M. Self-organized formation of polarized cortical tissues from escs and its active manipulation by extrinsic signals. Cell Stem Cell. (2008) 3:519–32. 10.1016/j.stem.2008.09.002 18983967

[42] Sato T Vries RG Snippert HJ Van De Wetering M Barker N Stange DE. Single Lgr5 stem cells build crypt-villus structures *in vitro* without a mesenchymal niche. Nature. (2009) 459:262–5. 10.1038/nature07935 19329995

[43] von Furstenberg RJ Li J Stolarchuk C Feder R Campbell A Kruger L. Porcine esophageal submucosal gland culture model shows capacity for proliferation and differentiation. Cmgh. (2017) 4:385–404. 10.1016/j.jcmgh.2017.07.005 28936470 PMC5602779

[44] Powell RH Behnke MS. WRN conditioned media is sufficient for *in vitro* propagation of intestinal organoids from large farm and small companion animals. Biol Open. (2017) 6:698–705. 10.1242/bio.021717 28347989 PMC5450310

[45] Gonzalez LM Williamson I Piedrahita JA Blikslager AT Magness ST. Cell lineage identification and stem cell culture in a porcine model for the study of intestinal epithelial regeneration. PLoS ONE. (2013) 8:e0066465. 10.1371/journal.pone.0066465 23840480 PMC3696067

[46] van der Hee B Madsen O Vervoort J Smidt H Wells JM. Congruence of transcription programs in adult stem cell-derived jejunum organoids and original tissue during long-term culture. Front Cell Dev Biol. (2020) 8:e00375. 10.3389/fcell.2020.00375 32714922 PMC7343960

[47] van der Hee B Loonen LMP Taverne N Taverne-Thiele JJ Smidt H Wells JM. Optimized procedures for generating an enhanced, near physiological 2D culture system from porcine intestinal organoids. Stem Cell Res. (2018) 28:165–71. 10.1016/j.scr.2018.02.013 29499500

[48] Sharbati J Hanisch C Pieper R Einspanier R Sharbati S. Small molecule and RNAi induced phenotype transition of expanded and primary colonic epithelial cells. Sci Rep. (2015) 5:12681. 10.1038/srep12681 26223582 PMC4519788

[49] Engevik AC Coutts AW Kaji I Rodriguez P Ongaratto F Saqui-Salces M. Editing myosin VB gene to create porcine model of microvillus inclusion disease, with microvillus-lined inclusions and alterations in sodium transporters. Gastroenterology. (2020) 158:2236–49.e9. 10.1053/j.gastro.2020.02.034 32112796 PMC7282982

[50] Li L Xue M Fu F Yin L Feng L Liu P. Ifn-lambda 3 mediates antiviral protection against porcine epidemic diarrhea virus by inducing a distinct antiviral transcript profile in porcine intestinal epithelia. Front Immunol. (2019) 10:e0239. 10.3389/fimmu.2019.02394 31681286 PMC6811514

[51] Li L Fu F Guo S Wang H He X Xue M. Porcine intestinal enteroids: a new model for studying enteric coronavirus porcine epidemic diarrhea virus infection and the host innate response. J Virol. (2019) 93:e01682–18. 10.1128/JVI.01682-18 30541861 PMC6384061

[52] Koltes DA Gabler NK. Characterization of porcine intestinal enteroid cultures under a lipopolysaccharide challenge. J Anim Sci. (2016) 94:335–9. 10.2527/jas.2015-9793

[53] Yin L Chen J Li L Guo S Xue M Zhang J. Aminopeptidase N expression, not interferon responses, determines the intestinal segmental tropism of porcine deltacoronavirus. J Virol. (2020) 94:e00480–20. 10.1128/JVI.00480-20 32376622 PMC7343211

[54] Luo H Zheng J Chen Y Wang T Zhang Z Shan Y. Utility evaluation of porcine enteroids as PDCoV infection model *in vitro*. Front Microbiol. (2020) 11:e00821. 10.3389/fmicb.2020.00821 32390999 PMC7191032

[55] Li XG Zhu M Chen MX Fan HB Fu HL Zhou JY. Acute exposure to deoxynivalenol inhibits porcine enteroid activity *via* suppression of the Wnt/β-catenin pathway. Toxicol Lett. (2019) 305:19–31. 10.1016/j.toxlet.2019.01.008 30690062

[56] Zhou J-y Huang D-g Zhu M Gao C-q Yan H-c Li X-g. Wnt/β-catenin-mediated heat exposure inhibits intestinal epithelial cell proliferation and stem cell expansion through endoplasmic reticulum stress. J Cell Physiol. (2020) 235:5613–27. 10.1002/jcp.29492 31960439

[57] Zhu M Qin YC Gao CQ Yan HC Wang XQ. L-Glutamate drives porcine intestinal epithelial renewal by increasing stem cell activity: *via* upregulation of the EGFR-ERK-mTORC1 pathway. Food Funct. (2020) 11:2714–24. 10.1039/C9FO03065D 32163057

[58] Hoffmann P Schnepel N Langeheine M Kunnemann K Grassl GA Brehm R. Intestinal organoid-based 2D monolayers mimic physiological and pathophysiological properties of the pig intestine. PLoS ONE. (2021) 16:e0256143. 10.1371/journal.pone.0256143 34424915 PMC8382199

[59] Zhu M Qin YC Gao CQ Yan HC Li XG Wang XQ. Extracellular glutamate-induced mTORC1 activation *via* the IR/IRS/PI3K/Akt pathway enhances the expansion of porcine intestinal stem cells. J Agric Food Chem. (2019) 67:9510–21. 10.1021/acs.jafc.9b03626 31382738

[60] Derricott H Luu LFWY Hartley CS Johnston LJ Armstrong SD Randle N. Coombes JL. Developing a 3D intestinal epithelium model for livestock species. Cell Tissue Res. (2019) 375:409–24. 10.1007/s00441-018-2924-9 30259138 PMC6373265

[61] Khalil HA Lei NY Brinkley G Scott A Wang J Kar UK. A novel culture system for adult porcine intestinal crypts. Cell Tissue Res. (2016) 365:123–34. 10.1007/s00441-016-2367-0 26928041 PMC4919165

[62] Li Y Yang N Chen J Huang X. Next-generation porcine intestinal organoids: an apical-out organoid model for swine enteric virus infection and immune response investigations. J Virol. (2020) 94:e01006–20. 10.1128/JVI.01006-20 32796075 PMC7565635

[63] Wang Z Li J Wang Y Wang L Yin Y Yin L. Dietary vitamin A affects growth performance, intestinal development, and functions in weaned piglets by affecting intestinal stem cells. J Anim Sci. (2020) 98:1–11. 10.1093/jas/skaa020 31955210 PMC7023621

[64] Resende TP Medida RL Vannucci FA Saqui-Salces M Gebhart C. Evaluation of swine enteroids as *in vitro* models for Lawsonia intracellularis infection. J Anim Sci. (2020) 98:1–5. 10.1093/jas/skaa011 31943029 PMC7007770

[65] Vila MF Trudeau MP Hung YT Zeng Z Urriola PE Shurson GC. Dietary fiber sources and non-starch polysaccharide-degrading enzymes modify mucin expression and the immune profile of the swine ileum. PLoS ONE. (2018) 13:e0207196. 10.1371/journal.pone.0207196 30408134 PMC6224153

[66] Stewart AS Freund JM Blikslager AT Gonzalez LM. Intestinal stem cell isolation and culture in a porcine model of segmental small intestinal ischemia. J Vis Exp. (2018) 135:e57647. 10.3791/57647 29863654 PMC6101266

[67] Callesen MM Árnadóttir SS Lyskjær I Ørntoft MBW Høyer S Dagnæs-Hansen F. A genetically inducible porcine model of intestinal cancer. Mol Oncol. (2017) 11:1616–29. 10.1002/1878-0261.12136 28881081 PMC5664002

[68] Zarei K Stroik MR Gansemer ND Thurman AL Ostedgaard LS Ernst SE. Early pathogenesis of cystic fibrosis gallbladder disease in a porcine model. Lab Investig. (2020) 100:1388–99. 10.1038/s41374-020-0474-8 32719544 PMC7578062

[69] Sakib S Uchida A Valenzuela-Leon P Yu Y Valli-Pulaski H Orwig K. Formation of organotypic testicular organoids in microwell culture. Biol Reprod. (2019) 100:1648–60. 10.1093/biolre/ioz053 30927418 PMC7302515

[70] Cham TC Ibtisham F Fayaz MA Honaramooz A. Generation of a highly biomimetic organoid, including vasculature, resembling the native immature testis tissue. Cells. (2021) 10:696. 10.3390/cells10071696 34359871 PMC8305979

[71] Hamilton CA Young R Jayaraman S Sehgal A Paxton E Thomson S. Development of *in vitro* enteroids derived from bovine small intestinal crypts. Vet Res. (2018) 49:54. 10.1186/s13567-018-0547-5 29970174 PMC6029049

[72] Töpfer E Pasotti A Telopoulou A Italiani P Boraschi D Ewart MA. Bovine colon organoids: from 3D bioprinting to cryopreserved multi-well screening platforms. Toxicol Vitr. (2019) 61:104606. 10.1016/j.tiv.2019.104606 31344400

[73] Fitzgerald SF Beckett AE Palarea-Albaladejo J McAteer S Shaaban S Morgan J. Shiga toxin sub-type 2a increases the efficiency of Escherichia coli O157 transmission between animals and restricts epithelial regeneration in bovine enteroids. PLoS Pathog. (2019) 15:e1008003. 10.1371/journal.ppat.1008003 31581229 PMC6776261

[74] Alfajaro MM Kim J-Y Barbé L Cho E-H Park J-G Soliman M. Dual recognition of sialic acid and αGal epitopes by the VP8^*^ domains of the bovine rotavirus G6P[5] WC3 and of its mono-reassortant G4P[5] RotaTeq vaccine strains. J Virol. (2019) 93:e00941–19. 10.1128/JVI.00941-19 31243129 PMC6714814

[75] Martignani E Accornero P Miretti S Baratta M. Bovine mammary organoids: a model to study epithelial mammary cells. Methods Mol Biol. (2018) 1817:137–44. 10.1007/978-1-4939-8600-2_14 29959710

[76] Bourdon G Cadoret V Charpigny G Couturier-Tarrade A Dalbies-Tran R Flores MJ. Progress and challenges in developing organoids in farm animal species for the study of reproduction and their applications to reproductive biotechnologies. Vet Res. (2021) 52:42. 10.1186/s13567-020-00891-w 33691745 PMC7944619

[77] Liu M Yu W Jin J Ma M An T Nie Y. Copper promotes sheep pancreatic duct organoid growth by activation of an antioxidant protein 1-dependent MEK-ERK pathway. Am J Physiol - Cell Physiol. (2020) 318:C806–16. 10.1152/ajpcell.00509.2019 32130071

[78] Stewart AS Freund JM Gonzalez LM. Advanced three-dimensional culture of equine intestinal epithelial stem cells. Equine Vet J. (2018) 50:241–8. 10.1111/evj.12734 28792626 PMC5796842

[79] Thompson RE Johnson AK Dini P Turco MY Prado TM Premanandan C. Hormone-responsive organoids from domestic mare and endangered Przewalski's horse endometrium. Reproduction. (2020) 160:819–31. 10.1530/REP-20-0266 33112764

[80] Pierzchalska M Grabacka M Michalik M Zyla K Pierzchalski P. Prostaglandin E2 supports growth of chicken embryo intestinal organoids in Matrigel matrix. Biotechniques. (2012) 52:307–15. 10.2144/0000113851 22578123

[81] Li J Li J Zhang SY Li RX Lin X Mi YL. Culture and characterization of chicken small intestinal crypts. Poult Sci. (2018) 97:1536–43. 10.3382/ps/pey010 29509914

[82] Panek M Grabacka M Pierzchalska M. The formation of intestinal organoids in a hanging drop culture. Cytotechnology. (2018) 70:1085–95. 10.1007/s10616-018-0194-8 29372467 PMC6021282

[83] Pierzchalska M Panek M Czyrnek M Grabacka M. The three-dimensional culture of epithelial organoids derived from embryonic chicken intestine. In: Turksen K, editor>. Organoids. Methods in Molecular Biology, Vol 1576. New York, NY: Humana Press (2016). 10.1007/7651_2016_15 27787775

[84] Acharya M Arsi K Donoghue AM Liyanage R Rath NC. Production and characterization of avian crypt-villus enteroids and the effect of chemicals. BMC Vet Res. (2020) 16:179. 10.1186/s12917-020-02397-1 32503669 PMC7275437

[85] Pierzchalska M Panek M Czyrnek M Gielicz A Mickowska B Grabacka M. Probiotic Lactobacillus acidophilus bacteria or synthetic TLR2 agonist boost the growth of chicken embryo intestinal organoids in cultures comprising epithelial cells and myofibroblasts. Comp Immunol Microbiol Infect Dis. (2017) 53:7–18. 10.1016/j.cimid.2017.06.002 28750869

[86] Kramer N Pratscher B Meneses AMC Tschulenk W Walter I Swoboda A. Generation of differentiating and long-living intestinal organoids reflecting the cellular diversity of canine intestine. Cells. (2020) 9:822. 10.3390/cells9040822 32231153 PMC7226743

[87] Chandra L Borcherding DC Kingsbury D Atherly T Ambrosini YM Bourgois-Mochel A. Derivation of adult canine intestinal organoids for translational research in gastroenterology. BMC Biol. (2019) 17:33. 10.1186/s12915-019-0652-6 30975131 PMC6460554

[88] Nantasanti S Spee B Kruitwagen HS Chen C Geijsen N Oosterhoff LA. Disease modeling and gene therapy of copper storage disease in canine hepatic organoids. Stem Cell Reports. (2015) 5:895–907. 10.1016/j.stemcr.2015.09.002 26455412 PMC4649105

[89] Kruitwagen HS Oosterhoff LA van Wolferen ME Chen C Nantasanti Assawarachan S Schneeberger K. Long-term survival of transplanted autologous canine liver organoids in a COMMD1-deficient dog model of metabolic liver disease. Cells. (2020) 9:410. 10.3390/cells9020410 32053895 PMC7072637

[90] Chen TC Neupane M Chien SJ Chuang FR Crawford RB Kaminski NE. Characterization of adult canine kidney epithelial stem cells that give rise to dome-forming tubular cells. Stem Cells Dev. (2019) 28:1424–33. 10.1089/scd.2019.0049 31495275

[91] Elbadawy M Usui T Mori T Tsunedomi R Hazama S Nabeta R. Establishment of a novel experimental model for muscle-invasive bladder cancer using a dog bladder cancer organoid culture. Cancer Sci. (2019) 110:2806–21. 10.1111/cas.14118 31254429 PMC6726682

[92] Abugomaa A Elbadawy M Yamanaka M Goto Y Hayashi K Mori T. Establishment of 25D organoid culture model using 3D bladder cancer organoid culture. Sci Rep. (2020) 10:9393. 10.1038/s41598-020-66229-w 32523078 PMC7287130

[93] Wiener DJ Basak O Asra P Boonekamp KE Kretzschmar K Papaspyropoulos A. Establishment and characterization of a canine keratinocyte organoid culture system. Vet Dermatol. (2018) 29:375–e126. 10.1111/vde.12541 29963730

[94] Wiener DJ Studer IC Brunner MAT Hermann A Vincenti S Zhang M. Characterization of canine epidermal organoid cultures by immunohistochemical analysis and quantitative PCR. Vet Dermatol. (2021) 32:179–e44. 10.1111/vde.12914 33165993

[95] Jankovic J Dettwiler M Fernández MG Tièche E Hahn K April-Monn S. Validation of immunohistochemistry for canine proteins involved in thyroid iodine uptake and their expression in canine follicular cell thyroid carcinomas (FTCs) and FTC-derived organoids. Vet Pathol. (2021) 58:1172–80. 10.1177/03009858211018813 34056980

[96] Tekes G Ehmann R Boulant S Stanifer ML. Development of feline ileum- and colon-derived organoids and their potential use to support feline coronavirus infection. Cells. (2020) 9:2085. 10.3390/cells9092085 32932592 PMC7563363

[97] Mussard E Pouzet C Helies V Pascal G Fourre S Cherbuy C. Culture of rabbit caecum organoids by reconstituting the intestinal stem cell niche *in vitro* with pharmacological inhibitors or L-WRN conditioned medium. Stem Cell Res. (2020) 48:101980. 10.1016/j.scr.2020.101980 32920507

[98] Kardia E Frese M Smertina E Strive T Zeng XL Estes M. Culture and differentiation of rabbit intestinal organoids and organoid-derived cell monolayers. Sci Rep. (2021) 11:5401. 10.1038/s41598-021-84774-w 33686141 PMC7940483

[99] Yum LK Agaisse H. Mechanisms of bacillary dysentery: lessons learnt from infant rabbits. Gut Microbes. (2020) 11:597–602. 10.1080/19490976.2019.1667726 31570038 PMC7524307

[100] VanDussen KL Marinshaw JM Shaikh N Miyoshi H Moon C Tarr PI. Development of an enhanced human gastrointestinal epithelial culture system to facilitate patient-based assays. Gut. (2015) 64:911–20. 10.1136/gutjnl-2013-306651 25007816 PMC4305344

[101] Sato T Stange DE Ferrante M Vries RGJ Van Es JH Van Den Brink S. Long-term expansion of epithelial organoids from human colon, adenoma, adenocarcinoma, and Barrett's epithelium. Gastroenterology. (2011) 141:1762–72. 10.1053/j.gastro.2011.07.050 21889923

[102] Huch M Dorrell C Boj SF Van Es JH Li VSW Van De Wetering M. *In vitro* expansion of single Lgr5 + liver stem cells induced by Wnt-driven regeneration. Nature. (2013) 494:247–50. 10.1038/nature11826 23354049 PMC3634804

[103] Huch M Gehart H Van Boxtel R Hamer K Blokzijl F Verstegen MMA. Long-term culture of genome-stable bipotent stem cells from adult human liver. Cell. (2015) 160:299–312. 10.1016/j.cell.2014.11.050 25533785 PMC4313365

[104] Nantasanti S De Bruin A Rothuizen J. Concise review: organoids are a powerful tool for the study of liver disease and personalized treatment design in humans and animals. Stem Cells Trans Med. (2016) 5:325–30. 10.5966/sctm.2015-0152 26798060 PMC4807664

[105] Huch M Bonfanti P Boj SF Sato T Loomans CJM Van De Wetering M. Unlimited in vitro expansion of adult bi-potent pancreas progenitors through the Lgr5/R-spondin axis. EMBO J. (2013) 32:2708–21. 10.1038/emboj.2013.204 24045232 PMC3801438

[106] Little MH Combes AN. Kidney organoids: accurate models or fortunate accidents. Genes Dev. (2019) 33:1319–45. 10.1101/gad.329573.119 31575677 PMC6771389

[107] Khoshdel-Rad N Ahmadi A Moghadasali R. Kidney organoids: current knowledge and future directions. Cell Tissue Res. (2022) 387:207–24. 10.1007/s00441-021-03565-x 35088178 PMC8794626

[108] Janssen DAW Geutjes PJ Odenthal J Van Kuppevelt TH Schalken JA Feitz WFJ. A new, straightforward *ex vivo* organoid bladder mucosal model for preclinical research. J Urol. (2013) 190:341–9. 10.1016/j.juro.2012.12.103 23306090

[109] Mullenders J de Jongh E Brousali A Roosen M Blom JPA Begthel H. Mouse and human urothelial cancer organoids: a tool for bladder cancer research. Proc Natl Acad Sci USA. (2019) 116:4567–74. 10.1073/pnas.1803595116 30787188 PMC6410883

[110] Sharma K Thacker VV Dhar N Clapés Cabrer M Dubois A Signorino-Gelo F. Early invasion of the bladder wall by solitary bacteria protects UPEC from antibiotics and neutrophil swarms in an organoid model. Cell Rep. (2021) 36:e109351. 10.1016/j.celrep.2021.109351 34289360

[111] Boretto M Cox B Noben M Hendriks N Fassbender A Roose H. Development of organoids from mouse and human endometrium showing endometrial epithelium physiology and long-term expandability. Development. (2017) 144:1775–86. 10.1242/dev.148478 28442471

[112] Turco MY Gardner L Hughes J Cindrova-Davies T Gomez MJ Farrell L. Long-term, hormone-responsive organoid cultures of human endometrium in a chemically defined medium. Nat Cell Biol. (2017) 19:568–77. 10.1038/ncb3516 28394884 PMC5410172

[113] Hillreiner M Müller NI Koch HM Schmautz C Küster B Pfaffl MW. Establishment of a 3D cell culture model of primary bovine mammary epithelial cells extracted from fresh milk. Vitr Cell Dev Biol - Anim. (2017) 53:706–20. 10.1007/s11626-017-0169-7 28643224

[114] Le Jan C Bellaton C Greenland T Mornex JF. Mammary transmission of caprine arthritis encephalitis virus: A 3D model for *in vitro* study. Reprod Nutr Dev. (2005) 45:513–23. 10.1051/rnd:2005035 16045898

[115] Abdelmegeed SM Mohammed S. Canine mammary tumors as a model for human disease (Review). Oncol Lett. (2018) 15:8195–205. 10.3892/ol.2018.8411 29928319 PMC6004712

[116] Souci L Denesvre C. 3D skin models in domestic animals. Vet Res. (2021) 52:1–15. 10.1186/s13567-020-00888-5 33588939 PMC7885517

[117] Herkenne C Naik A Kalia YN Hadgraft J Guy RH. Pig ear skin *ex vivo* as a model for *in vivo* dermatopharmacokinetic studies in man. Pharm Res. (2006) 23:1850–6. 10.1007/s11095-006-9011-8 16841197

[118] Marsella R De Benedetto A. Atopic dermatitis in animals and people: an update and comparative review. Vet Sci. (2017) 4:37. 10.3390/vetsci4030037 29056696 PMC5644664

[119] Saito Y Onishi N Takami H Seishima R Inoue H Hirata Y. Development of a functional thyroid model based on an organoid culture system. Biochem Biophys Res Commun. (2018) 497:783–9. 10.1016/j.bbrc.2018.02.154 29470983

[120] McHenry CR Phitayakorn R. Follicular adenoma and carcinoma of the thyroid gland. Oncologist. (2011) 16:585–93. 10.1634/theoncologist.2010-0405 21482585 PMC3228182

[121] Sang Y Miller LC Nelli RK Giménez-Lirola LG. Harness organoid models for virological studies in animals: a cross-species perspective. Front Microbiol. (2021) 12:e725074. 10.3389/fmicb.2021.725074 34603253 PMC8481363

[122] Zhou J Li C Liu X Chiu MC Zhao X Wang D. Infection of bat and human intestinal organoids by SARS-CoV-2. Nat Med. (2020) 26:1077–83. 10.1038/s41591-020-0912-6 32405028

[123] Kelly AM Ferguson JD Galligan DT Salman M Osburn BI. One health, food security, and veterinary medicine. J Am Vet Med Assoc. (2013) 242:739–43. 10.2460/javma.242.6.739 23445280

[124] Liu Q Wang HY. Porcine enteric coronaviruses: an updated overview of the pathogenesis, prevalence, and diagnosis. Vet Res Commun. (2021) 45:75–86. 10.1007/s11259-021-09808-0 34251560 PMC8273569

[125] Wensink GE Elias SG Mullenders J Koopman M Boj SF Kranenburg OW. Patient-derived organoids as a predictive biomarker for treatment response in cancer patients. npj Precis. Onc. (2021) 5:30. 10.1038/s41698-021-00168-1 33846504 PMC8042051

[126] Minkler S Lucien F Kimber MJ Sahoo DK Bourgois-Mochel A Musser M. Emerging roles of urine-derived components for the management of bladder cancer: one man's trash is another man's treasure. Cancers. (2021) 13:1–13. 10.3390/cancers13030422 33498666 PMC7865365

[127] Creevy KE Akey JM Kaeberlein M Promislow DEL Barnett BG Benton B. An open science study of ageing in companion dogs. Nature. (2022) 602:51–7. 10.1038/s41586-021-04282-9 35110758 PMC8940555

[128] Jensen C Teng Y. Is it time to start transitioning from 2D to 3D cell culture? Front Mol Biosci. (2020) 7:33. 10.3389/fmolb.2020.00033 32211418 PMC7067892

[129] Leung E Kim JE Askarian-Amiri M Finlay GJ Baguley BC. Evidence for the existence of triple-negative variants in the MCF-7 breast cancer cell population. Biomed Res Int. (2014) 2014:836769. 10.1155/2014/836769 24724101 PMC3960520

[130] Ben-David U Siranosian B Ha G Tang H Oren Y Hinohara K. Genetic and transcriptional evolution alters cancer cell line drug response. Nature. (2018) 560:325–30. 10.1038/s41586-018-0409-3 30089904 PMC6522222

[131] Gjorevski N Ranga A Lutolf MP. Bioengineering approaches to guide stem cell-based organogenesis. Development. (2014) 141:1794–804. 10.1242/dev.101048 24757002

[132] Oliveira B Çerag Yahya A Novarino G. Modeling cell-cell interactions in the brain using cerebral organoids. Brain Res. (2019) 1724:146458. 10.1016/j.brainres.2019.146458 31521639

[133] Dekkers JF Wiegerinck CL De Jonge HR Bronsveld I Janssens HM De Winter-De Groot KM. A functional CFTR assay using primary cystic fibrosis intestinal organoids. Nat Med. (2013) 19:939–45. 10.1038/nm.3201 23727931

[134] Sondorp LHJ Ogundipe VML Groen AH Kelder W Kemper A Links TP. Patient-derived papillary thyroid cancer organoids for radioactive iodine refractory screening. Cancers. (2020) 12:3212. 10.3390/cancers12113212 33142750 PMC7692469

[135] Bigorgne AE Farin HF Lemoine R Mahlaoui N Lambert N Gil M. TTC7A mutations disrupt intestinal epithelial apicobasal polarity. J Clin Invest. (2014) 124:328–37. 10.1172/JCI71471 24292712 PMC3871247

[136] Wiegerinck CL Janecke AR Schneeberger K Vogel GF Van Haaften-Visser DY Escher JC. Loss of syntaxin 3 causes variant microvillus inclusion disease. Gastroenterology. (2014) 147:65–8.e10. 10.1053/j.gastro.2014.04.002 24726755

[137] Yan HHN Siu HC Law S Ho SL Yue SSK Tsui WY. A comprehensive human gastric cancer organoid biobank captures tumor subtype heterogeneity and enables therapeutic screening. Cell Stem Cell. (2018) 23:882–97.e11. 10.1016/j.stem.2018.09.016 30344100

[138] Van De Wetering M Francies HE Francis JM Bounova G Iorio F Pronk A. Prospective derivation of a living organoid biobank of colorectal cancer patients. Cell. (2015) 161:933–45. 10.1016/j.cell.2015.03.053 25957691 PMC6428276

[139] Jacob F Salinas RD Zhang DY Nguyen PTT Schnoll JG Wong SZH. A patient-derived glioblastoma organoid model and biobank recapitulates inter- and intra-tumoral heterogeneity. Cell. (2020) 180:188–204.e22. 10.1016/j.cell.2019.11.036 31883794 PMC7556703

[140] Sachs N de Ligt J Kopper O Gogola E Bounova G Weeber F. A living biobank of breast cancer organoids captures disease heterogeneity. Cell. (2018) 172:373–86.e10. 10.1016/j.cell.2017.11.010 29224780

[141] Yang H Sun L Liu M Mao Y. Patient-derived organoids: a promising model for personalized cancer treatment. Gastroenterol Rep. (2018) 6:243–5. 10.1093/gastro/goy040 30430011 PMC6225812

[142] Tran F Klein C Arlt A Imm S Knappe E Simmons A. Stem cells and organoid technology in precision medicine in inflammation: are we there yet? Front Immunol. (2020) 11:e573562. 10.3389/fimmu.2020.573562 33408713 PMC7779798

[143] Barker N Huch M Kujala P van de Wetering M Snippert HJ van Es JH. Lgr5+ve stem cells drive self-renewal in the stomach and build long-lived gastric units *in vitro*. Cell Stem Cell. (2010) 6:25–36. 10.1016/j.stem.2009.11.013 20085740

[144] Lu YC Fu DJ An D Chiu A Schwartz R Nikitin AY. Scalable production and cryostorage of organoids using core–shell decoupled hydrogel capsules. Adv Biosyst. (2017) 1:1700165. 10.1002/adbi.201700165 29607405 PMC5870136

[145] Han SH Shim S Kim MJ Shin HY Jang WS Lee SJ. Long-term culture-induced phenotypic difference and efficient cryopreservation of small intestinal organoids by treatment timing of Rho kinase inhibitor. World J Gastroenterol. (2017) 23:964–75. 10.3748/wjg.v23.i6.964 28246470 PMC5311106

[146] Miyoshi H Stappenbeck TS. *In vitro* expansion and genetic modification of gastrointestinal stem cells in spheroid culture. Nat Protoc. (2013) 8:2471–82. 10.1038/nprot.2013.153 24232249 PMC3969856

[147] Yui S Nakamura T Sato T Nemoto Y Mizutani T Zheng X. Functional engraftment of colon epithelium expanded *in vitro* from a single adult Lgr5 + stem cell. Nat Med. (2012) 18:618–23. 10.1038/nm.2695 22406745

[148] Lõhmussaar K Oka R Espejo Valle-Inclan J Smits MHH Wardak H Korving J. Patient-derived organoids model cervical tissue dynamics and viral oncogenesis in cervical cancer. Cell Stem Cell. (2021) 28:1380–96.e6. 10.1016/j.stem.2021.03.012 33852917

[149] Wilson SS Bromme BA Holly MK Wiens ME Gounder AP Sul Y. Alpha-defensin-dependent enhancement of enteric viral infection. PLoS Pathog. (2017) 13:e1006446. 10.1371/journal.ppat.1006446 28622386 PMC5489213

[150] Yokoi Y Nakamura K Yoneda T Kikuchi M Sugimoto R Shimizu Y. Paneth cell granule dynamics on secretory responses to bacterial stimuli in enteroids. Sci Rep. (2019) 9:2710. 10.1038/s41598-019-39610-7 30804449 PMC6389922

[151] García-Rodríguez I Sridhar A Pajkrt D Wolthers KC. Put some guts into it: intestinal organoid models to study viral infection. Viruses. (2020) 12:1288. 10.3390/v12111288 33187072 PMC7697248

[152] Puzan M Hosic S Ghio C Koppes A. Enteric nervous system regulation of intestinal stem cell differentiation and epithelial monolayer function. Sci Rep. (2018) 8:6313. 10.1038/s41598-018-24768-3 29679034 PMC5910425

[153] Braverman J Yilmaz ÖH. From 3D organoids back to 2D enteroids. Dev Cell. (2018) 44:533–4. 10.1016/j.devcel.2018.02.016 29533766

[154] Park GS Park MH Shin W Zhao C Sheikh S Oh SJ. Emulating host-microbiome ecosystem of human gastrointestinal tract *in vitro*. Stem Cell Rev Reports. (2017) 13:321–34. 10.1007/s12015-017-9739-z 28488235

[155] Shin W Wu A Massidda MW Foster C Thomas N Lee DW. A robust longitudinal co-culture of obligate anaerobic gut microbiome with human intestinal epithelium in an anoxic-oxic interface-on-a-chip. Front Bioeng Biotechnol. (2019) 7:13. 10.3389/fbioe.2019.00013 30792981 PMC6374617

[156] Bhatia SN Ingber DE. Microfluidic organs-on-chips. Nat Biotechnol. (2014) 32:760–72. 10.1038/nbt.2989 25093883

[157] Kim HJ Li H Collins JJ Ingber DE. Contributions of microbiome and mechanical deformation to intestinal bacterial overgrowth and inflammation in a human gut-on-a-chip. Proc Natl Acad Sci USA. (2016) 113:E7–15. 10.1073/pnas.1522193112 26668389 PMC4711860

[158] Huh D Matthews BD Mammoto A Montoya-Zavala M Yuan Hsin H Ingber DE. Reconstituting organ-level lung functions on a chip. Science. (2010) 328:1662–8. 10.1126/science.1188302 20576885 PMC8335790

[159] Shin YC Shin W Koh D Wu A Ambrosini YM Min S. Three-dimensional regeneration of patient-derived intestinal organoid epithelium in a physiodynamic mucosal interface-on-a-chip. Micromachines. (2020) 11:663. 10.3390/mi11070663 32645991 PMC7408321

[160] Bhise NS Manoharan V Massa S Tamayol A Ghaderi M Miscuglio M. A liver-on-a-chip platform with bioprinted hepatic spheroids. Biofabrication. (2016) 8:014101. 10.1088/1758-5090/8/1/014101 26756674

[161] Wang Y Wang L Zhu Y Qin J. Human brain organoid-on-a-chip to model prenatal nicotine exposure. Lab Chip. (2018) 18:851–60. 10.1039/C7LC01084B 29437173

[162] Wang Y Wang L Guo Y Zhu Y Qin J. Engineering stem cell-derived 3D brain organoids in a perfusable organ-on-a-chip system. RSC Adv. (2018) 8:1677–85. 10.1039/C7RA11714K 35540867 PMC9077091

[163] Zhang YS Aleman J Arneri A Bersini S Shin SR Dokmeci MR. Bioprinting 3D microfibrous scaffolds for engineering endothelialized myocardium and heart-on-a-chip. Biomaterials. (2016) 110:45–59. 10.1016/j.biomaterials.2016.09.003 27710832 PMC5198581

[164] Agarwal A Goss JA Cho A McCain ML Parker KK. Microfluidic heart on a chip for higher throughput pharmacological studies. Lab Chip. (2013) 13:3599–608. 10.1039/c3lc50350j 23807141 PMC3786400

[165] Wilmer MJ Ng CP Lanz HL Vulto P Suter-Dick L Masereeuw R. Kidney-on-a-chip technology for drug-induced nephrotoxicity screening. Trends Biotechnol. (2016) 34:156–70. 10.1016/j.tibtech.2015.11.001 26708346

[166] Achberger K Probst C Haderspeck JC Bolz S Rogal J Chuchuy J. Merging organoid and organ-on-a-chip technology to generate complex multi-layer tissue models in a human retina-on-a-chip platform. Elife. (2019) 8:e46188. 10.7554/eLife.46188 31451149 PMC6777939

[167] Dodson KH Echevarria FD Li D Sappington RM Edd JF. Retina-on-a-chip: a microfluidic platform for point access signaling studies. Biomed Microdev. (2015) 17:114. 10.1007/s10544-015-0019-x 26559199 PMC4707151

[168] Kasendra M Tovaglieri A Sontheimer-Phelps A Jalili-Firoozinezhad S Bein A Chalkiadaki A. Development of a primary human Small Intestine-on-a-Chip using biopsy-derived organoids. Sci Rep. (2018) 8:2871. 10.1038/s41598-018-21201-7 29440725 PMC5811607

[169] Tang H Abouleila Y Si L Ortega-Prieto AM Mummery CL Ingber DE. Human organs-on-chips for virology. Trends Microbiol. (2020) 28:934–46. 10.1016/j.tim.2020.06.005 32674988 PMC7357975

[170] Fabre KM Livingston C Tagle DA. Organs-on-chips (microphysiological systems): tools to expedite efficacy and toxicity testing in human tissue. Exp Biol Med. (2014) 239:1073–7. 10.1177/1535370214538916 24962171

[171] Esch EW Bahinski A Huh D. Organs-on-chips at the frontiers of drug discovery. Nat Rev Drug Discov. (2015) 14:248–60. 10.1038/nrd4539 25792263 PMC4826389

[172] Peck RW Hinojosa CD Hamilton GA. Organs-on-chips in clinical pharmacology: putting the patient into the center of treatment selection and drug development. Clin Pharmacol Ther. (2020) 107:181–5. 10.1002/cpt.1688 31758803 PMC6977308

[173] Ronaldson-Bouchard K Vunjak-Novakovic G. Organs-on-a-chip: a fast track for engineered human tissues in drug development. Cell Stem Cell. (2018) 22:310–24. 10.1016/j.stem.2018.02.011 29499151 PMC5837068

[174] de Almeida Monteiro Melo Ferraz M Nagashima JB Venzac B Le Gac S Songsasen N A. dog oviduct-on-a-chip model of serous tubal intraepithelial carcinoma. Sci Rep. (2020) 10:1575. 10.1038/s41598-020-58507-4 32005926 PMC6994655

[175] Ferraz MAMM Henning HHW Costa PF Malda J Melchels FP Wubbolts R. Improved bovine embryo production in an oviduct-on-a-chip system: prevention of poly-spermic fertilization and parthenogenic activation. Lab Chip. (2017) 17:905–16. 10.1039/C6LC01566B 28194463

