# Supplementary material for: Farm and Companion Animal Organoid Models in Translational Research: A Powerful Tool to Bridge the Gap Between Mice and Humans
Source: Front Med Technol. 2022 May 12;4:895379. doi: 10.3389/fmedt.2022.895379 (PMC9133531; doi:10.3389/fmedt.2022.895379)
Supplement: Supplementary file 1 [file Table_1.pdf]

Supplementary Material

1 Supplementary Data

Supplementary Table 1. Summary of studies related to 3D organoids in humans and animals.

System	Tissue	Human	Mouse	Pig	Dog	Cattle	Chicken	Rabbit	Cat	Horse	Sheep	NHP
Neurological	Brain	✓[1]	✓[1]									
	Retina	✓[2]	✓[3]									
	Inner ear	✓[4]	✓[5]									
Cardiovascular	Heart	✓[6]	✓[7]									
	Blood vessel	✓[8]										
Respiratory	Lung	✓[9]	✓[10]									
Digestive	Salivary gland	✓[11]	✓[12]									
	Esophagus	✓[13]	✓[14]	✓[15]								
	Stomach	✓[16]	✓[17]									
	Intestine (segment unknown)		✓[18]	✓[19,20]			✓[21–25]					
	Duodenum	✓[26]	✓[27]	✓[28–33]	✓[34,35]		✓[25]	✓[36]				
	Jejunum	✓[37]	✓[38]	✓[28,29,31–33,39–49]	✓[34,35]	✓[39,50]	✓[51]	✓[36]		✓[52]		
	Ileum	✓[38]	✓[53]	✓[29,31–33,54–57]	✓[34,54,58]	✓[54,59–61]		✓[36]	✓[54,62]	✓[54]	✓[54]	
	Cecum		✓[63]				✓[54]	✓[64]				
	Colon	✓[38]	✓[65]	✓[29,32,66,67]	✓[34,35,68]	✓[69]			✓[62]			
	Liver and Gallbladder	✓[70]	✓[71]	✓[72]	✓[73,74]				✓[75,76]			
	Pancreas	✓[77]	✓[78]								✓[79]	
Urinary	Kidney	✓[80]	✓[80]		✓[81]							

	Bladder	✓[82]	✓[82]		✓[83,84]			
Reproductive	Ovary and Oviduct	✓[85]	✓[86]			✓[87]		
	Uterus	✓[88]	✓[88]				✓[89]	
	Placenta	✓[90]						
	Mammary gland	✓[91]	✓[92]			✓[93]		
	Testis	✓[94]	✓[94]	✓[94,95]				✓[94]
	Prostate	✓[96]	✓[96]		✓[97]			
Endocrine	Thyroid	✓[98]	✓[98]		✓[99]			
Dermatological	Skin	✓[100]	✓[101]		✓[102,103]			
Musculoskeletal	Bone	✓[104]	✓[105]					
	Skeletal muscle	✓[106]						

NHP = non-human primate.

References for Supplementary Table 1

1. Lancaster MA, Renner M, Martin CA, Wenzel D, Bicknell LS, Hurles ME, et al. Cerebral organoids model human brain development and microcephaly. *Nature*. (2013) 501:373–9. doi: 10.1038/nature12517

2. Cowan CS, Renner M, De Gennaro M, Gross-Scherf B, Goldblum D, Hou Y, et al. Cell Types of the Human Retina and Its Organoids at Single-Cell Resolution. *Cell*. (2020) 182:1623-1640.e34. doi: 10.1016/j.cell.2020.08.013

3. Völkner M, Kurth T, Schor J, Ebner LJA, Bardtke L, Kavak C, et al. Mouse Retinal Organoid Growth and Maintenance in Longer-Term Culture. *Front Cell Dev Biol*. (2021) 9:645704. doi: 10.3389/fcell.2021.645704

4. Koehler KR, Nie J, Longworth-Mills E, Liu XP, Lee J, Holt JR, et al. Generation of inner ear organoids containing functional hair cells from human pluripotent stem cells. *Nat Biotechnol*. (2017) 35:583–9. doi: 10.1038/nbt.3840

5. Chang SY, Carpena NT, Mun S, Jung JY, Chung PS, Shim H, et al. Enhanced Inner-Ear Organoid Formation from Mouse Embryonic Stem Cells by Photobiomodulation. *Mol Ther - Methods Clin Dev*. (2020) 17:556–67. doi: 10.1016/j.omtm.2020.03.010

6. Mills RJ, Humphrey SJ, Fortuna PRJ, Lor M, Foster SR, Quaife-Ryan GA, et al. BET inhibition blocks inflammation-induced cardiac dysfunction and SARS-CoV-2 infection. *Cell*. (2021) 184:2167-2182.e22. doi: 10.1016/j.cell.2021.03.026

7. Lee J, Sutani A, Kaneko R, Takeuchi J, Sasano T, Kohda T, et al. In vitro generation of functional murine heart organoids via FGF4 and extracellular matrix. *Nat Commun*. (2020) 11:4283. doi: 10.1038/s41467-020-18031-5

8. Monteil V, Kwon H, Prado P, Hagelkrüys A, Wimmer RA, Stahl M, et al. Inhibition of SARS-CoV-2 Infections in Engineered Human Tissues Using Clinical-Grade Soluble Human ACE2. *Cell*. (2020) 181:905-913.e7. doi: 10.1016/j.cell.2020.04.004

9. Han Y, Duan X, Yang L, Nilsson-Payant BE, Wang P, Duan F, et al. Identification of SARS-CoV-2 inhibitors using lung and colonic organoids. *Nature*. (2021) 589:270–5. doi: 10.1038/s41586-020-2901-9

10. Hai J, Zhang H, Zhou J, Wu Z, Chen T, Papadopoulos E, et al. Generation of genetically engineered mouse lung organoid models for squamous cell lung cancers allows for the study of combinatorial immunotherapy. *Clin Cancer Res*. (2020) 26:3431–42. doi: 10.1158/1078-0432.CCR-19-1627

11. Sui Y, Zhang S, Li Y, Zhang X, Hu W, Feng Y, et al. Generation of functional salivary gland tissue from human submandibular gland stem/progenitor cells. *Stem Cell Res Ther*. (2020) 11:127. doi: 10.1186/s13287-020-01628-4

12. Athwal H, Lombaert I. 3D Organoid Formation from the Murine Salivary Gland Cell Line SIMS. *Bio-Protocol*. (2019) 9: e3386. doi: 10.21769/BioProtoc.3386

13. Trisno SL, Philo KED, McCracken KW, Catá EM, Ruiz-Torres S, Rankin SA, et al. Esophageal Organoids from Human Pluripotent Stem Cells Delineate Sox2 Functions during Esophageal Specification. *Cell Stem Cell*. (2018) 23:501-515.e7. doi: 10.1016/j.stem.2018.08.008

14. Zheng B, Ko KP, Fang X, Wang X, Zhang J, Jun S, et al. A new murine esophageal organoid culture method and organoid-based model of esophageal squamous cell neoplasia. *iScience*. (2021) 24:103440. doi: 10.1016/j.isci.2021.103440

15. von Furstenberg RJ, Li J, Stolarchuk C, Feder R, Campbell A, Kruger L, et al. Porcine Esophageal Submucosal Gland Culture Model Shows Capacity for Proliferation and Differentiation. *Cmgh*. (2017) 4:385–404. doi: 10.1016/j.jcmgh.2017.07.005

16. Lo YH, Kolahi KS, Du Y, Chang CY, Krokhotin A, Nair A, et al. A crispr/cas9-engineered arid1a-deficient human gastric cancer organoid model reveals essential and nonessential modes of oncogenic transformation. *Cancer Discov*. (2021) 11:1562–81. doi: 10.1158/2159-8290.CD-20-1109

17. Chakrabarti J, Holokai L, Syu LJ, Steele N, Chang J, Dlugosz A, et al. Mouse-Derived Gastric Organoid and Immune Cell Co-culture for the Study of the Tumor Microenvironment. *Methods Mol Biol*. (2018) 1817:157–68. doi: 10.1007/978-1-4939-8600-2_16

18. Haber AL, Biton M, Rogel N, Herbst RH, Shekhar K, Smillie C, et al. A single-cell survey of the small intestinal epithelium. *Nature*. (2017) 551:333–9. doi: 10.1038/nature24489

19. Engevik AC, Coutts AW, Kaji I, Rodriguez P, Ongaratto F, Saqui-Salces M, et al. Editing Myosin VB Gene to Create Porcine Model of Microvillus Inclusion Disease, With Microvillus-Lined Inclusions and Alterations in Sodium Transporters. *Gastroenterology*. (2020) 158:2236-2249.e9. doi: 10.1053/j.gastro.2020.02.034

20. Li L, Xue M, Fu F, Yin L, Feng L, Liu P. Ifn-lambda 3 mediates antiviral protection against porcine epidemic diarrhea virus by inducing a distinct antiviral transcript profile in porcine intestinal epithelia. *Front Immunol*. (2019) 10:2394. doi: 10.3389/fimmu.2019.02394

21. Acharya M, Arsi K, Donoghue AM, Liyanage R, Rath NC. Production and characterization of avian crypt-villus enteroids and the effect of chemicals. *BMC Vet Res*. (2020) 16:179. doi: 10.1186/s12917-020-02397-1

22. Pierzchalska M, Grabacka M, Michalik M, Zyla K, Pierzchalski P. Prostaglandin E2 supports growth of chicken embryo intestinal organoids in Matrigel matrix. *Biotechniques.* (2012) 52:307–15. doi: 10.2144/0000113851

23. Pierzchalska M, Panek M, Czyrnek M, Grabacka M. The Three-Dimensional Culture of Epithelial Organoids Derived from Embryonic Chicken Intestine. In: *Methods in Molecular Biology*. (2016). p. 135–44. Turksen K (ed) Humana, New York. doi: 10.1007/7651_2016_15

24. Panek M, Grabacka M, Pierzchalska M. The formation of intestinal organoids in a hanging drop culture. *Cytotechnology*. (2018) 70:1085–95. doi: 10.1007/s10616-018-0194-8

25. Pierzchalska M, Panek M, Czyrnek M, Gielicz A, Mickowska B, Grabacka M. Probiotic Lactobacillus acidophilus bacteria or synthetic TLR2 agonist boost the growth of chicken embryo intestinal organoids in cultures comprising epithelial cells and myofibroblasts. *Comp Immunol Microbiol Infect Dis*. (2017) 53:7–18. doi: 10.1016/j.cimid.2017.06.002

26. Yamashita T, Inui T, Yokota J, Kawakami K, Morinaga G, Takatani M, et al. Monolayer platform using human biopsy-derived duodenal organoids for pharmaceutical research. *Mol Ther - Methods Clin Dev*. (2021) 22:263–78. doi: 10.1016/j.omtm.2021.05.005

27. Kar SK, Van Der Hee B, Loonen LMP, Taverne N, Taverne-Thiele JJ, Schokker D, et al. Effects of undigested protein-rich ingredients on polarised small intestinal organoid monolayers. *J Anim Sci Biotechnol*. (2020) 11:51. doi: 10.1186/s40104-020-00443-4

28. Gonzalez LM, Williamson I, Piedrahita JA, Blikslager AT, Magness ST. Cell Lineage Identification and Stem Cell Culture in a Porcine Model for the Study of Intestinal Epithelial Regeneration. *PLoS One*. (2013) 8:e66465. doi: 10.1371/journal.pone.0066465

29. Li L, Fu F, Guo S, Wang H, He X, Xue M, et al. Porcine Intestinal Enteroids: a New Model for Studying Enteric Coronavirus Porcine Epidemic Diarrhea Virus Infection and the Host Innate Response. *J Virol*. (2019) 93:e01682-18. doi: 10.1128/JVI.01682-18

30. Koltes DA, Gabler NK. Characterization of porcine intestinal enteroid cultures under a lipopolysaccharide challenge. *J Anim Sci*. (2016) 94:335–9. doi:10.2527/jas2015-9793

31. Vermeire B, Gonzalez LM, Jansens RJJ, Cox E, Devriendt B. Porcine small intestinal organoids as a model to explore ETEC-host interactions in the gut. *Vet Res*. (2021) 52:94. doi: 10.1186/s13567-021-00961-7

32. Yin L, Chen J, Li L, Guo S, Xue M, Zhang J, et al. Aminopeptidase N Expression, Not Interferon Responses, Determines the Intestinal Segmental Tropism of Porcine Deltacoronavirus. *J Virol*. (2020) 94:e00480-20. doi: 10.1128/JVI.00480-20

33. Luo H, Zheng J, Chen Y, Wang T, Zhang Z, Shan Y, et al. Utility Evaluation of Porcine Enteroids as PDCoV Infection Model in vitro. *Front Microbiol*. (2020) 11:821. doi: 10.3389/fmicb.2020.00821

34. Chandra L, Borcherding DC, Kingsbury D, Atherly T, Ambrosini YM, Bourgois-Mochel A, et al. Derivation of adult canine intestinal organoids for translational research in gastroenterology. *BMC Biol*. (2019) 17:33. doi: 10.1186/s12915-019-0652-6

35. Kramer N, Pratscher B, Meneses AMC, Tschulenk W, Walter I, Swoboda A, et al. Generation of Differentiating and Long-Living Intestinal Organoids Reflecting the Cellular Diversity of Canine Intestine. *Cells*. (2020) 9:822. doi: 10.3390/cells9040822

36. Kardia E, Frese M, Smertina E, Strive T, Zeng XL, Estes M, et al. Culture and differentiation of rabbit intestinal organoids and organoid-derived cell monolayers. *Sci Rep*. (2021) 11:5401. doi: 10.1038/s41598-021-84774-w

37. Engevik MA, Danhof HA, Chang-Graham AL, Spinler JK, Engevik KA, Herrmann B, et al. Human intestinal enteroids as a model of Clostridioides difficile-induced enteritis. *Am J Physiol - Gastrointest Liver Physiol*. (2020) 318:G870–88. doi: 10.1152/ajpgi.00045.2020

38. Rao X, Tang P, Li Y, Fu G, Chen S, Xu X, et al. CBP/P300 Inhibitors Mitigate Radiation-Induced GI Syndrome by Promoting Intestinal Stem Cell-Mediated Crypt Regeneration. *Int J Radiat Oncol Biol Phys*. (2021) 110:1210–21. doi: 10.1016/j.ijrobp.2021.01.046

39. Derricott, H; Luu LF, W.Y.; Hartley, C.S.; Johnston, L.J.; Armstrong, S.D.; Randle, N.; Duckworth, C.A.; Campbell, B.J.; Wastling, J.M.; Coombes JL. Developing a 3D intestinal epithelium model for livestock species. *Cell Tissue Res*. (2019) 375:409–24. doi: 10.1007/s00441-018-2924-9

40. Khalil, HA; Lei, NY; Brinkley, G; Scott, A; Wang, J; Kar, UK; Jabaji, ZB; Lewis, M; Martin, MG; Dunn, JCY; Stelzner M. A Novel Culture System for Adult Porcine Intestinal Crypts. *Cell Tissue Res*. (2016) 365:123–34. doi: 10.1007/s00441-016-2367-0

41. van der Hee B, Madsen O, Vervoort J, Smidt H, Wells JM. Congruence of Transcription Programs in Adult Stem Cell-Derived Jejunum Organoids and Original Tissue During Long-Term Culture. *Front Cell Dev Biol*. (2020) 8:375. doi: 10.3389/fcell.2020.00375

42. Li Y, Yang N, Chen J, Huang X, Zhang N, Yang S, et al. Next-Generation Porcine Intestinal Organoids: an Apical-Out Organoid Model for Swine Enteric Virus Infection and Immune Response Investigations. *J Virol*. (2020) 94:e01006-20. doi: 10.1128/JVI.01006-20

43. Stewart AS, Freund JM, Blikslager AT, Gonzalez LM. Intestinal stem cell isolation and culture in a porcine model of segmental small intestinal ischemia. *J Vis Exp*. (2018) 135:e57647. doi: 10.3791/57647

44. Wang Z, Li J, Wang Y, Wang L, Yin Y, Yin L, et al. Dietary vitamin A affects growth performance, intestinal development, and functions in weaned piglets by affecting intestinal stem cells. *J Anim Sci*. (2020) 98:skaa020. doi: 10.1093/jas/skaa020

45. Li XG, Zhu M, Chen MX, Fan HB, Fu HL, Zhou JY, et al. Acute exposure to deoxynivalenol inhibits porcine enteroid activity via suppression of the Wnt/β-catenin pathway. *Toxicol Lett*. (2019) 305:19–31. doi: 10.1016/j.toxlet.2019.01.008

46. Zhou J yi, Huang D gui, Zhu M, Gao C qi, Yan H chao, Li X guang, et al. Wnt/β-catenin-mediated heat exposure inhibits intestinal epithelial cell proliferation and stem cell expansion through endoplasmic reticulum stress. *J Cell Physiol*. (2020) 235:5613–27. doi: 10.1002/jcp.29492

47. Zhu M, Qin YC, Gao CQ, Yan HC, Wang XQ. L-Glutamate drives porcine intestinal epithelial renewal by increasing stem cell activity: Via upregulation of the EGFR-ERK-mTORC1 pathway. *Food Funct*. (2020) 11:2714–24. doi: 10.1039/c9fo03065d

48. Hoffmann P, Schnepel N, Langeheine M, Kunnemann K, Grassl GA, Brehm R, et al. Intestinal organoid-based 2D monolayers mimic physiological and pathophysiological properties of the pig intestine. *PLoS One*. (2021) 16:e0256143. doi: 10.1371/journal.pone.0256143

49. Zhu M, Qin YC, Gao CQ, Yan HC, Li XG, Wang XQ. Extracellular Glutamate-Induced mTORC1 Activation via the IR/IRS/PI3K/Akt Pathway Enhances the Expansion of Porcine Intestinal Stem Cells. *J Agric Food Chem*. (2019) 67:9510–21. doi: 10.1021/acs.jafc.9b03626

50. Lee BR, Yang H, Lee SI, Haq I, Ock SA, Wi H, et al. Robust three-dimensional (3d) expansion of bovine intestinal organoids: An in vitro model as a potential alternative to an in vivo system. *Animals*. (2021) 11:2115. doi: 10.3390/ani11072115

51. Li J, Li J, Zhang SY, Li RX, Lin X, Mi YL, et al. Culture and characterization of chicken small intestinal crypts. *Poult Sci*. (2018) 97:1536–43. doi: 10.3382/ps/pey010

52. Stewart AS, Freund JM, Gonzalez LM. Advanced three-dimensional culture of equine intestinal epithelial stem cells. *Equine Vet J*. (2018) 50:241–8. doi: 10.1111/evj.12734

53. Deng F, Zhao BC, Yang X, Lin Z Bin, Sun QS, Wang YF, et al. The gut microbiota metabolite capsiate promotes Gpx4 expression by activating TRPV1 to inhibit intestinal ischemia reperfusion-induced ferroptosis. *Gut Microbes*. (2021) 13: e1902719. doi: 10.1080/19490976.2021.1902719

54. Powell RH, Behnke MS. WRN conditioned media is sufficient for in vitro propagation of intestinal organoids from large farm and small companion animals. *Biol Open*. (2017) 6:698–705. doi: 10.1242/bio.021717

55. van der Hee B, Loonen LMP, Taverne N, Taverne-Thiele JJ, Smidt H, Wells JM. Optimized procedures for generating an enhanced, near physiological 2D culture system from porcine intestinal organoids. *Stem Cell Res*. (2018) 28:165–71. doi: 10.1016/j.scr.2018.02.013

56. Resende TP, Medida RL, Vannucci FA, Saqui-Salces M, Gebhart C. Evaluation of swine enteroids as in vitro models for Lawsonia intracellularis infection. *J Anim Sci*. (2020) 98:skaa011. doi: 10.1093/jas/skaa011

57. Vila MF, Trudeau MP, Hung YT, Zeng Z, Urriola PE, Shurson GC, et al. Dietary fiber sources and non-starch polysaccharide-degrading enzymes modify mucin expression and the immune profile of the swine ileum. *PLoS One*. (2018) 13:e0207196. doi: 10.1371/journal.pone.0207196

58. Agopian VG, Chen DC, Avansino JR, Stelzner M. Intestinal stem cell organoid transplantation generates neomucosa in dogs. *J Gastrointest Surg*. (2009) 13:971–82. doi: 10.1007/s11605-009-0806-x

59. Hamilton CA, Young R, Jayaraman S, Sehgal A, Paxton E, Thomson S, et al. Development of in vitro enteroids derived from bovine small intestinal crypts. *Vet Res*. (2018) 49:54. doi: 10.1186/s13567-018-0547-5

60. Fitzgerald SF, Beckett AE, Palarea-Albaladejo J, McAteer S, Shaaban S, Morgan J, et al. Shiga toxin sub-type 2a increases the efficiency of Escherichia coli O157 transmission between animals and restricts epithelial regeneration in bovine enteroids. *PLoS Pathog*. (2019) 15:e1008003. doi: 10.1371/journal.ppat.1008003

61. Alfajaro MM, Kim J-Y, Barbé L, Cho E-H, Park J-G, Soliman M, et al. Dual Recognition of Sialic Acid and αGal Epitopes by the VP8* Domains of the Bovine Rotavirus G6P[5] WC3 and of Its Mono-reassortant G4P[5] RotaTeq Vaccine Strains. *J Virol*. (2019) 93:e00941-19. doi: 10.1128/JVI.00941-19

62. Tekes G, Ehmann R, Boulant S, Stanifer ML. Development of Feline Ileum- and Colon-Derived Organoids and Their Potential Use to Support Feline Coronavirus Infection. *Cells*. (2020) 9:2085. doi: 10.3390/cells9092085

63. Duque-Correa MA, Schreiber F, Rodgers FH, Goulding D, Forrest S, White R, et al. Development of caecaloids to study host–pathogen interactions: new insights into immunoregulatory functions of Trichuris muris extracellular vesicles in the caecum. Int J Parasitol. (2020) 50:707–18. doi: 10.1016/j.ijpara.2020.06.001

64. Mussard E, Pouzet C, Helies V, Pascal G, Fourre S, Cherbuy C, et al. Culture of rabbit caecum organoids by reconstituting the intestinal stem cell niche in vitro with pharmacological inhibitors or L-WRN conditioned medium. *Stem Cell Res*. (2020) 48:101980. doi: 10.1016/j.scr.2020.101980

65. Sato T, Stange DE, Ferrante M, Vries RGJ, Van Es JH, Van Den Brink S, et al. Long-term expansion of epithelial organoids from human colon, adenoma, adenocarcinoma, and Barrett’s epithelium. Gastroenterology. (2011) 141:1762–72. doi: 10.1053/j.gastro.2011.07.050

66. Sharbati J, Hanisch C, Pieper R, Einspanier R, Sharbati S. Small molecule and RNAi induced phenotype transition of expanded and primary colonic epithelial cells. *Sci Rep*. (2015) 5:12681. doi: 10.1038/srep12681

67. Callesen MM, Árnadóttir SS, Lyskjær I, Ørntoft MBW, Høyer S, Dagnæs-Hansen F, et al. A genetically inducible porcine model of intestinal cancer. *Mol Oncol*. (2017) 11:1616–29. doi: 10.1002/1878-0261.12136

68. Ambrosini YM, Park Y, Jergens AE, Shin W, Min S, Atherly T, et al. Recapitulation of the accessible interface of biopsy-derived canine intestinal organoids to study epithelial-luminal interactions. *PLoS One*. (2020) 15:e0231423. doi: 10.1371/journal.pone.0231423

69. Töpfer E, Pasotti A, Telopoulou A, Italiani P, Boraschi D, Ewart MA, et al. Bovine colon organoids: From 3D bioprinting to cryopreserved multi-well screening platforms. *Toxicol Vitr*. (2019) 61:104606. doi: 10.1016/j.tiv.2019.104606

70. Huch M, Gehart H, Van Boxtel R, Hamer K, Blokzijl F, Verstegen MMA, et al. Long-term culture of genome-stable bipotent stem cells from adult human liver. *Cell*. (2015) 160:299–312. doi: 10.1016/j.cell.2014.11.050

71. Huch M, Dorrell C, Boj SF, Van Es JH, Li VSW, Van De Wetering M, et al. In vitro expansion of single Lgr5 + liver stem cells induced by Wnt-driven regeneration. *Nature*. (2013) 494:247–50. doi: 10.1038/nature11826

72. Zarei K, Stroik MR, Gansemer ND, Thurman AL, Ostedgaard LS, Ernst SE, et al. Early pathogenesis of cystic fibrosis gallbladder disease in a porcine model. *Lab Investig*. (2020) 100:1388–99. doi: 10.1038/s41374-020-0474-8

73. Nantasanti S, Spee B, Kruitwagen HS, Chen C, Geijsen N, Oosterhoff LA, et al. Disease modeling and gene therapy of copper storage disease in canine hepatic organoids. *Stem Cell Reports*. (2015) 5:895–907. doi: 10.1016/j.stemcr.2015.09.002

74. Kruitwagen HS, Oosterhoff LA, van Wolferen ME, Chen C, Nantasanti Assawarachan S, Schneeberger K, et al. Long-Term Survival of Transplanted Autologous Canine Liver Organoids in a COMMD1-Deficient Dog Model of Metabolic Liver Disease. *Cells*. (2020) 9:410. doi: 10.3390/cells9020410

75. Kruitwagen HS, Oosterhoff LA, Vernooij IGWH, Schrall IM, van Wolferen ME, Bannink F, et al. Long-Term Adult Feline Liver Organoid Cultures for Disease Modeling of Hepatic Steatosis. *Stem Cell Reports*. (2017) 8:822–30. doi: 10.1016/j.stemcr.2017.02.015

76. Haaker MW, Kruitwagen HS, Vaandrager AB, Houweling M, Penning LC, Molenaar MR, et al. Identification of potential drugs for treatment of hepatic lipidosis in cats using an in vitro feline liver organoid system. *J Vet Intern Med*. (2020) 34:132–8. doi: 10.1111/jvim.15670

77. Boj SF, Hwang C Il, Baker LA, Chio IIC, Engle DD, Corbo V, et al. Organoid models of human and mouse ductal pancreatic cancer. *Cell*. (2015) 160:324–38. doi: 10.1016/j.cell.2014.12.021

78. Huch M, Bonfanti P, Boj SF, Sato T, Loomans CJM, Van De Wetering M, et al. Unlimited in vitro expansion of adult bi-potent pancreas progenitors through the Lgr5/R-spondin axis. *EMBO J*. (2013) 32:2708–21. doi: 10.1038/emboj.2013.204

79. Liu M, Yu W, Jin J, Ma M, An T, Nie Y, et al. Copper promotes sheep pancreatic duct organoid growth by activation of an antioxidant protein 1-dependent MEK-ERK pathway. *Am J Physiol - Cell Physiol*. (2020) 318:C806–16. doi: 10.1152/ajpcell.00509.2019

80. Schutgens F, Rookmaaker MB, Margaritis T, Rios A, Ammerlaan C, Jansen J, et al. Tubuloids derived from human adult kidney and urine for personalized disease modeling. *Nat Biotechnol*. (2019) 37:303–13. doi: 10.1038/s41587-019-0048-8

81. Chen TC, Neupane M, Chien SJ, Chuang FR, Crawford RB, Kaminski NE, et al. Characterization of Adult Canine Kidney Epithelial Stem Cells That Give Rise to Dome-Forming Tubular Cells. *Stem Cells Dev*. (2019) 28:1424–33. doi: 10.1089/scd.2019.0049

82. Mullenders J, de Jongh E, Brousali A, Roosen M, Blom JPA, Begthel H, et al. Mouse and human urothelial cancer organoids: A tool for bladder cancer research. *Proc Natl Acad Sci USA*. (2019) 116:4567–74. doi: 10.1073/pnas.1803595116

83. Elbadawy M, Usui T, Mori T, Tsunedomi R, Hazama S, Nabeta R, et al. Establishment of a novel experimental model for muscle-invasive bladder cancer using a dog bladder cancer organoid culture. *Cancer Sci*. (2019) 110:2806–21. doi: 10.1111/cas.14118

84. Abugomaa A, Elbadawy M, Yamanaka M, Goto Y, Hayashi K, Mori T, et al. Establishment of 2.5D organoid culture model using 3D bladder cancer organoid culture. *Sci Rep*. (2020) 10:9393. doi: 10.1038/s41598-020-66229-w

85. Kopper O, de Witte CJ, Lõhmussaar K, Valle-Inclan JE, Hami N, Kester L, et al. An organoid platform for ovarian cancer captures intra- and interpatient heterogeneity. *Nat Med*. (2019) 25:838–49. doi: 10.1038/s41591-019-0422-6

86. Xie Y, Park ES, Xiang D, Li Z. Long-term organoid culture reveals enrichment of organoid-forming epithelial cells in the fimbrial portion of mouse fallopian tube. *Stem Cell Res*. (2018) 32:51–60. doi: 10.1016/j.scr.2018.08.021

87. Bourdon G, Cadoret V, Charpigny G, Couturier-Tarrade A, Dalbies-Tran R, Flores MJ, et al. Progress and challenges in developing organoids in farm animal species for the study of reproduction and their applications to reproductive biotechnologies. *Vet Res*. (2021) 52:42. doi: 10.1186/s13567-020-00891-w

88. Boretto M, Cox B, Noben M, Hendriks N, Fassbender A, Roose H, et al. Development of organoids from mouse and human endometrium showing endometrial epithelium physiology and long-term expandability. *Dev*. (2017) 144:1775–86. doi: 10.1242/dev.148478

89. Thompson RE, Johnson AK, Dini P, Turco MY, Prado TM, Premanandan C, et al. Hormone-responsive organoids from domestic mare and endangered Przewalski's horse endometrium. *Reproduction*. (2020) 160:819–31. doi: 10.1530/REP-20-0266

90. Turco MY, Gardner L, Kay RG, Hamilton RS, Prater M, Hollinshead MS, et al. Trophoblast organoids as a model for maternal–fetal interactions during human placentation. *Nature*. (2018) 564:263–267. doi: 10.1038/s41586-018-0753-3

91. Qu Y, Han B, Gao B, Bose S, Gong Y, Wawrowsky K, et al. Differentiation of Human Induced Pluripotent Stem Cells to Mammary-like Organoids. *Stem Cell Reports*. (2017) 8:205–15. doi: 10.1016/j.stemcr.2016.12.023

92. Shackleton M, Vaillant F, Simpson KJ, Stingl J, Smyth GK, Asselin-Labat ML, et al. Generation of a functional mammary gland from a single stem cell. *Nature*. (2006) 439:84–8. doi: 10.1038/nature04372

93. Martignani E, Accornero P, Miretti S, Baratta M. Bovine Mammary Organoids: A Model to Study Epithelial Mammary Cells. *Methods Mol Biol*. (2018) 1817:137–44. doi: 10.1007/978-1-4939-8600-2_14

94. Sakib S, Uchida A, Valenzuela-Leon P, Yu Y, Valli-Pulaski H, Orwig K, et al. Formation of organotypic testicular organoids in microwell culture. *Biol Reprod*. (2019) 100:1648–60. doi: 10.1093/biolre/ioz053

95. Cham TC, Ibtisham F, Fayaz MA, Honaramooz A. Generation of a highly biomimetic organoid, including vasculature, resembling the native immature testis tissue. *Cells*. (2021) 10:1696. doi: 10.3390/cells10071696

96. Drost J, Karthaus WR, Gao D, Driehuis E, Sawyers CL, Chen Y, et al. Organoid culture systems for prostate epithelial and cancer tissue. *Nat Protoc*. (2016) 11:347–58. doi: 10.1038/nprot.2016.006

97. Usui T, Sakurai M, Nishikawa S, Umata K, Nemoto Y, Haraguchi T, et al. Establishment of a dog primary prostate cancer organoid using the urine cancer stem cells. *Cancer Sci*. (2017) 108:2383–92. doi: 10.1111/cas.13418

98. Ogundipe VML, Groen AH, Hosper N, Nagle PWK, Hess J, Faber H, et al. Generation and Differentiation of Adult Tissue-Derived Human Thyroid Organoids. *Stem Cell Reports*. (2021) 16:913–25. doi: 10.1016/j.stemcr.2021.02.011

99. Jankovic J, Dettwiler M, Fernández MG, Tièche E, Hahn K, April-Monn S, et al. Validation of Immunohistochemistry for Canine Proteins Involved in Thyroid Iodine Uptake and Their Expression in Canine Follicular Cell Thyroid Carcinomas (FTCs) and FTC-Derived Organoids. *Vet Pathol*. (2021) 58:1172–80. doi: 10.1177/03009858211018813

100. Lee J, Rabbani CC, Gao H, Steinhart MR, Woodruff BM, Pflum ZE, et al. Hair-bearing human skin generated entirely from pluripotent stem cells. *Nature*. (2020) 582:399–404. doi: 10.1038/s41586-020-2352-3

101. Lei M, Schumacher LJ, Lai YC, Juan WT, Yeh CY, Wu P, et al. Self-organization process in newborn skin organoid formation inspires strategy to restore hair regeneration of adult cells. *Proc Natl Acad Sci USA*. (2017) 114:E7101–10. doi: 10.1073/pnas.1700475114

102. Wiener DJ, Basak O, Asra P, Boonekamp KE, Kretzschmar K, Papaspyropoulos A, et al. Establishment and characterization of a canine keratinocyte organoid culture system. *Vet Dermatol*. (2018) 29:375-e126. doi: 10.1111/vde.12541

103. Wiener DJ, Studer IC, Brunner MAT, Hermann A, Vincenti S, Zhang M, et al. Characterization of canine epidermal organoid cultures by immunohistochemical analysis and quantitative PCR. *Vet Dermatol*. (2021) 32:179-e44. doi: 10.1111/vde.12914

104. Pischon N, Zimmermann B, Bernimoulin JP, Hägewald S. Effects of an enamel matrix derivative on human osteoblasts and PDL cells grown in organoid cultures. *Oral Surgery, Oral Med Oral Pathol Oral Radiol Endodontology*. (2006) 102:551–7. doi: 10.1016/j.tripleo.2005.08.040.

105. Hägewald S, Pischon N, Jawor P, Bernimoulin JP, Zimmermann B. Effects of enamel matrix derivative on proliferation and differentiation of primary osteoblasts. *Oral Surgery, Oral Med Oral Pathol Oral Radiol Endodontology*. (2004) 98:243–9. doi: 10.1016/j.tripleo.2004.02.063

106. Maffioletti SM, Sarcar S, Henderson ABH, Mannhardt I, Pinton L, Moyle LA, et al. Three-Dimensional Human iPSC-Derived Artificial Skeletal Muscles Model Muscular Dystrophies and Enable Multilineage Tissue Engineering. *Cell Rep*. (2018) 23:899–908. doi: 10.1016/j.celrep.2018.03.091
